# Supplementary material for: Alcohol Exposure and Disease Associations: A Mendelian Randomization and Meta-Analysis on Weekly Consumption and Problematic Drinking
Source: Nutrients. 2024 May 17;16(10):1517. doi: 10.3390/nu16101517 (PMC11123792; doi:10.3390/nu16101517)
Supplement: Supplementary file 1 [file nutrients-16-01517-s001.zip › nutrients-2960036-supplementary.pdf]

Additional files

Alcohol Exposure and Disease Associations: A Mendelian Randomization and Meta-Analysis on Weekly Consumption and Problematic Drinking

Authors and institutions

Mengyao Li[1,2], Xuying Zhang[1,2], Kailei Chen[1,2], Yang Miao[1,2], Yaxin Xu[1,2], Yishuo Sun[1,2], Mengxian Jiang[1,2], Mengcao Liu[1,2], Yan Gao[1,2], Xiaoxia Xue[2,3], Xuelian Li[1,2*]

1, Key Laboratory of Environmental Stress and Chronic Disease Control & Prevention, China Medical University, Ministry of Education, Shenyang 110122, China.

2, Department of Epidemiology, School of Public Health, China Medical University, Shenyang 110122, China.

3, Science Experiment Center, China Medical University, No.77 Puhe Road, Shenyang North New Area, Shenyang 110122, China.

Corresponding author.

Xuelian Li

Department of Epidemiology, School of Public Health, China Medical University, Shenyang 110122, China.

Science Experiment Center, China Medical University, No.77 Puhe Road, Shenyang North New Area, Shenyang 110122, China.

E-mail address: **xlli@cmu.edu.cn**

Contents

Table S1. Literature Quality Scoring Criteria - 1 -
Table S2. Characteristics of Included Studies. - 2 -
Table S3. Single nucleotide polymorphism loci used as instrumental variables for alcohol consumption - 7 -
Table S4. Single nucleotide polymorphism loci used as instrumental variables for problematic alcohol use. - 12 -
Table S5. Characterization of outcome disease data - 14 -
Table S6. Mendelian randomization studies included in the meta-analyses of genetic liability to alcohol consumption in relation to Circulatory system diseases, Digestive system diseases, Nervous system diseases and mental and behavioral disorders, Neoplasms, and Other diseases. - 20 -
Table S7. Mendelian randomization studies included in the meta-analyses of genetic liability to problematic alcohol use in relation to Circulatory system diseases, Digestive system diseases, Nervous system diseases and mental and behavioral disorders, Neoplasms, and Other diseases. - 49 -
Table S8. MVMR analysis estimating the effect of alcohol consumption on disease, conditioning on smoking - 60 -
Table S9. The effects model was corrected using the B/H correction. - 62 -
Table S10. Search Strategies for PubMed, Embase, Web of Science, and Scopus Databases. - 64 -
Figure S1. Literature review and study design - 65 -
Figure S2. Genetic predictors of "alcohol consumption" and disease risk were summarized by IVW, WME, MR-Egger - 66 -
Figure S3. Genetic predictors of "problematic alcohol use" and disease risk were summarized by IVW, WME, MR-Egger - 67 -

Table S1. Literature Quality Scoring Criteria

Standard		score
Relevance assumption	Instrumental variable genome-wide association analysis was significant ($p < 5 \times 10^{-8}$) and instrumental variable F-statistic>10.	3
	Instrumental variable genome-wide association analysis was significant ($5 \times 10^{-8} < p < 0.05$) and instrumental variable F-statistic > 10.	2
	Not assessed or does not meet the above conditions	1
Independence assumption	Genetic independence of instrumental variables, and linkage disequilibrium effects were assessed by including populations of the same genetic background.	3
	Only the assumption is described and not fully assessed.	2
	Not described and not assessed	1
Exclusion-restriction assumption	Assessment of instrumental variables for pleiotropy, heterogeneity.	3
	Only the assumption is described and not fully assessed.	2
	Not described and not assessed	1
Problem of sample overlap	Mention the issue of overlapping samples of exposure and outcome use data and make relevant assessments.	2
	Not mentioned, assessed.	1
Statistical power	Mention and perform calculations	1
	Not mentioned, not calculated	0

Total score of 12; quality rating: low (0-4), medium (5-8), high (9-12).

Table S2. Characteristics of Included Studies.

Authors (Year)	PMID	Exposure	Source of exposure	Ethnicity of exosure	Outcome	Source of outcome	Ethnicity of outcome	Quality scores
Zhang (2022)	35334809	Alcohol consumption	30643251	European	Epilepsy	30531953	European	9
Andrews (2020)	31786126	Alcohol consumption	30643251	European	Alzheimer's Disease	28628103	European	11
Domínguez-Baleón (2021)	34234189	Alcohol consumption	30643251	European	Parkinson's Disease	31701892	European	9
Domenighetti (2022)	34633332	Alcohol consumption	30643251	European	Parkinson's Disease	JPND	European	9
Zhou (2022)	35102554	Alcohol consumption	30643251	European	Colorectal cancer	32638365	European	11
Larsson (2020)	32701947	Alcohol consumption	30643251	European	Colorectal cancer;<br>Pancreatic cancer;<br>Ovarian cancer;<br>Endometrial cancer;<br>Cervical cancer;<br>Prostate cancer;<br>Lung cancer;<br>Kidney cancer;<br>Brain cancer;<br>Head and neck cancer;	24880342<br>29059683<br>28346442<br>29892016<br>UK Biobank	European	10

					Malignant melanoma;<br>Non-Hodgkin lymphoma;<br>Leukemia;<br>Multiple myeloma			
Yuan (2023)	36727839	Alcohol consumption	30643251	European	Esophageal cancer；<br>Gastric cancer；<br>Cholecystitis；<br>Hepatocellular Carcinoma；<br>NAFLD；<br>Alcoholic Liver Disease；<br>Cirrhosis；<br>GERD；<br>Gastric Ulcer；<br>Duodenal Ulcer；<br>Gastric Ulcer；<br>Acute Pancreatitis；<br>Acute Appendicitis；<br>Diverticular Disease；<br>Crohn's Disease；<br>Ulcerative Colitis；<br>IBS	26192919<br>33893285<br>UK Biobank<br>FinnGen(r7)	European	11
Yuan (2022)	33418132	Alcohol consumption	30643251	European	Cholelithiasis	UK Biobank<br>FinnGen(r3)	European	9
Yuan (2022)	35488966	Alcohol consumption	30643251	European	NAFLD	UK Biobank<br>FinnGen	European	10

Yuan (2022)	35119566	Alcohol consumption	30643251	European	GERD	31527586	European	9
Georgiou (2021)	32628751	Alcohol consumption	30643251	European	Crohn's Disease; Ulcerative Colitis	28067908	European	12
Yuan (2021)	34187701	Alcohol consumption	30643251	European	Spontaneous abortion	UK Biobank	European	8
Rogne (2022)	35390318	Alcohol consumption	30643251	European	Ectopic pregnancy	UK Biobank FinnGen	European	7
Zhou (2022)	35708873	Alcohol consumption	30643251	European	Breast cancer	32424353	European	11
		Problematic alcohol use	32451486	European				
Wang (2022)	36309747	Alcohol consumption	30643251	European	Hyperplasia of prostate	UK Biobank FinnGen(r5)	European	9
Baumeister (2021)	34472130	Alcohol consumption	30643251	European	Chronic Periodontitis	31235808	European	10
Jiang (2021)	34239545	Alcohol consumption	30643251	European	Rheumatoid arthritis；Lupus erythematosus；Multiple Sclerosis	24390342 26502338 31604244	European	9
Yuan (2022)	35029599	Alcohol consumption	30643251	European	Migraine	27322543	European	9
Zhu (2023)	36862322	Alcohol consumption	30643251	European	Sepsis；Pneumonia；URIT；UTI	UK Biobank	European	9
Xiong (2022)	35904850	Alcohol consumption	30643251	European	Bladder cancer	UK Biobank FinnGen(r5)	European	10

Yuan (2022)	35013517	Alcohol consumption	30643251	European	Senile cataract	UK Biobank FinnGen(r4)	European	8
Kuan (2021)	34734970	Alcohol consumption	30643251	European	Age-related macular degeneration	26691988	European	11
Gormley (2020)	33247085	Alcohol consumption	30643251	European	Oral and oropharyngeal cancer	27749845	European	11
Oort (2020)	32682105	Alcohol consumption	30643251	European	Heart failure	31919418	European	12
Larsson (2020)	32367730	Alcohol consumption	30643251	European	Heart failure；Peripheral vascular disease；Stroke；Atrial fibrillation；Venous thromboembolism	UK Biobank	European	10
Hoek (2022)	35929454	Alcohol consumption	30643251	European	Peripheral vascular disease	31285632	European	10
Rosoff (2020)	33275596	Alcohol consumption	30643251	European	Coronary artery atherosclerosis；Myocardial infarction；Hypertension	UK Biobank FinnGen	European	9
Jiang (2020)	32540331	Alcohol consumption	30643251	European	Atrial fibrillation	30061737	European	10
Zhou (2022)	36003339	Alcohol consumption	30643251	European	Aortic aneurysm	UK Biobank FinnGen(r6)	European	9
Pasman (2020)	32634714	Alcohol consumption	30643251	European	Insomnia	UK Biobank	European	10

Yuan (2021)	34666504	Alcohol consumption	30643251	European	Varicose veins	UK Biobank FinnGen(r4)	European	12

PMID: PubMed database unique identifier; JPND: The EU Joint Programme–Neurodegenerative Disease Research; ADHD: Attention Deficit Hyperactivity Disorder syndrome; ALS: Amyotrophic lateral sclerosis; NAFLD: Nonalcoholic Fatty Liver Disease; GERD: Gastroesophageal reflux; IBS: irritable bowel syndrome; URIT: Acute upper respiratory tract infection; JPND: The EU joint programme–neurodegenerative disease; FinnGen: FinnGen database(https://www.finngen.fi/en); COVID-19 hg7: version 7 data published by the COVID-19 Host Genetics Program(https://www.covid19hg.org/results/r7/); UKB: The UK Biobank(https://www.ukbiobank.ac.uk/).

Table S3. Single nucleotide polymorphism loci used as instrumental variables for alcohol consumption.

SNP	EA	NEA	EAF	Beta	SE	P	F
rs61873510	T	G	0.306163	-0.0105	0.002	1.17E-08	27.56
rs74813718	G	C	0.0417495	-0.0189	0.003	1.97E-08	39.69
rs3133388	A	G	0.308151	-0.0157	0.002	2.85E-13	61.62
rs17613838	C	T	0.150099	0.0144	0.002	1.19E-10	51.84
rs1894071	G	A	0.845924	0.0168	0.002	7.60E-12	70.56
rs1699104	A	C	0.55169	0.0116	0.002	4.04E-08	33.64
rs11030084	T	C	0.190855	-0.0151	0.002	2.27E-11	57.00
rs7938022	C	T	0.714712	0.0121	0.002	4.25E-08	36.60
rs79175663	T	C	0.161034	0.0146	0.002	1.03E-09	53.29
rs2053980	C	A	0.333002	-0.0192	0.002	1.95E-18	92.16
rs10743083	G	A	0.815109	-0.015	0.002	1.64E-10	56.25
rs55863153	C	A	0.0178926	0.0316	0.005	2.48E-09	39.94
rs61929956	A	G	0.228628	-0.0118	0.002	1.98E-08	34.81
rs4463912	T	C	0.378728	-0.00992	0.002	2.77E-08	24.60
rs4761961	C	A	0.558648	-0.0108	0.002	5.19E-10	29.16
rs3809162	G	A	0.384692	0.0102	0.002	6.84E-09	26.01
rs56115085	T	C	0.145129	-0.0132	0.002	4.14E-08	43.56
rs11114780	T	C	0.222664	0.0132	0.002	1.60E-08	43.56
rs1821711	A	G	0.651093	-0.0101	0.002	2.13E-08	25.50
rs6538292	G	A	0.616302	-0.0107	0.002	2.12E-09	28.62
rs9670378	C	A	0.591451	-0.0107	0.002	2.32E-08	28.62
rs1123285	G	C	0.348907	-0.0109	0.002	2.59E-09	29.70
rs55791516	G	T	0.751491	-0.0156	0.002	1.46E-11	60.84

rs28929474	T	C	0.0168986	-0.0489	0.006	3.28E-14	66.42
rs9745238	G	C	0.667992	-0.0132	0.002	1.38E-09	43.56
rs2414133	G	A	0.286282	0.0106	0.002	3.02E-08	28.09
rs2472297	T	C	0.214712	0.0111	0.002	2.34E-08	30.80
rs28616142	T	C	0.418489	0.0112	0.002	2.99E-10	31.36
rs17177078	T	C	0.0725646	-0.0263	0.004	4.96E-13	43.23
rs2650496	C	T	0.340954	-0.0173	0.002	3.04E-14	74.82
rs3814877	T	G	0.382704	-0.0111	0.002	5.64E-10	30.80
rs1558902	A	T	0.432406	-0.0117	0.002	6.42E-11	34.22
rs62044501	G	A	0.186879	-0.0125	0.002	3.48E-08	39.06
rs11075711	T	C	0.16501	-0.0134	0.002	1.31E-08	44.89
rs62052824	G	A	0.203777	0.0141	0.003	4.16E-08	22.09
rs113968734	A	C	0.134195	0.017	0.002	1.00E-11	72.25
rs11860773	C	T	0.184891	-0.0157	0.002	1.04E-12	61.62
rs13332432	G	C	0.293241	0.0129	0.002	1.64E-11	41.60
rs1971157	C	G	0.394632	0.00987	0.002	2.74E-08	24.35
rs55938136	G	A	0.238569	-0.023	0.002	1.22E-27	132.25
rs147431626	A	G	0.249503	-0.0294	0.002	7.52E-32	216.09
rs11078696	T	G	0.710736	0.02	0.003	4.29E-12	44.44
rs4130668	C	T	0.770378	0.0119	0.002	1.77E-08	35.40
rs148390057	T	C	0.439364	-0.0111	0.002	2.91E-08	30.80
rs4890444	G	C	0.374751	0.0112	0.002	1.89E-09	31.36
rs78308288	T	C	0.198807	0.0129	0.002	1.48E-08	41.60
rs72926972	A	C	0.0675944	-0.0173	0.003	4.96E-08	33.25
rs1942964	G	T	0.49503	-0.0107	0.002	3.89E-09	28.62
rs838145	A	G	0.581511	-0.0164	0.002	5.62E-21	67.24

rs12748460	T	C	0.188867	-0.0144	0.002	1.36E-09	51.84
rs10753661	A	G	0.738569	-0.0113	0.002	1.25E-09	31.92
rs6691053	T	C	0.218688	-0.0138	0.002	1.24E-10	47.61
rs823099	A	C	0.414513	0.0105	0.002	2.31E-09	27.56
rs12044012	A	G	0.360835	-0.0116	0.002	1.34E-09	33.64
rs6698883	T	C	0.899602	-0.0161	0.003	1.14E-08	28.80
rs12121630	A	G	0.149105	-0.0136	0.002	1.59E-08	46.24
rs2310752	A	G	0.428429	-0.00977	0.002	2.57E-08	23.86
rs6136465	A	G	0.407555	-0.00982	0.002	3.01E-08	24.11
rs35006396	G	A	0.228628	0.0128	0.002	2.82E-08	40.96
rs4502500	G	C	0.370775	-0.00995	0.002	2.07E-08	24.75
rs7284839	T	C	0.798211	0.0155	0.003	3.66E-08	26.69
rs17884691	A	G	0.232604	-0.0114	0.002	2.91E-08	32.49
rs76217384	G	A	0.208748	0.0136	0.002	3.37E-11	46.24
rs6715038	T	C	0.738569	0.0124	0.002	9.53E-09	38.44
rs1001129	A	G	0.812127	-0.0122	0.002	4.18E-08	37.21
rs1973044	T	C	0.817097	0.0133	0.002	1.31E-08	44.22
rs11125160	A	G	0.661034	0.0102	0.002	3.96E-08	26.01
rs1260326	C	T	0.589463	0.0254	0.002	3.16E-46	161.29
rs441413	G	T	0.0785288	-0.0211	0.003	1.26E-09	49.47
rs62136829	T	C	0.0377734	-0.0295	0.004	3.74E-11	54.39
rs570305	G	C	0.578529	0.0175	0.002	3.19E-20	76.56
rs1949613	T	G	0.709742	0.013	0.002	8.14E-11	42.25
rs7566031	T	C	0.302187	0.0139	0.002	3.61E-13	48.30
rs828867	A	G	0.522863	0.0103	0.002	3.44E-09	26.52
rs11692435	A	G	0.083499	0.0191	0.003	7.20E-11	40.53

rs147711594	T	G	0.0248509	-0.0298	0.005	2.91E-08	35.52
rs66652990	A	T	0.243539	-0.014	0.002	6.19E-09	49.00
rs6787172	G	T	0.537773	-0.0107	0.002	7.49E-10	28.62
rs7618629	G	A	0.767396	0.0122	0.002	8.02E-10	37.21
rs60026303	G	A	0.178926	0.0121	0.002	3.58E-08	36.60
rs10154865	T	C	0.225646	0.0206	0.002	2.14E-23	106.09
rs13135092	G	A	0.0854871	-0.0357	0.003	4.00E-26	141.61
rs57590313	A	C	0.175944	-0.0129	0.002	4.60E-08	41.60
rs7682731	G	A	0.827038	0.015	0.002	1.25E-09	56.25
rs9685410	A	T	0.814115	-0.0132	0.002	4.00E-09	43.56
rs111203819	G	T	0.485089	0.0101	0.002	2.41E-08	25.50
rs12646808	C	T	0.348907	-0.011	0.002	2.47E-09	30.25
rs11940694	G	A	0.611332	0.0279	0.002	9.51E-56	194.60
rs58352691	T	G	0.0675944	0.0233	0.003	2.40E-11	60.32
rs1229984	C	T	0.971173	0.193	0.005	1.17E-283	1489.96
rs1693439	G	A	0.950298	0.0193	0.003	2.83E-08	41.39
rs1789889	A	G	0.146123	0.0266	0.002	1.30E-32	176.89
rs284784	A	C	0.218688	-0.0119	0.002	1.13E-08	35.40
rs10491278	C	A	0.145129	-0.0135	0.002	2.96E-08	45.56
rs6886898	G	T	0.562624	-0.00951	0.002	4.51E-08	22.61
rs55872084	T	G	0.22664	0.012	0.002	4.68E-09	36.00
rs6899302	C	T	0.514911	-0.0104	0.002	1.70E-09	27.04
rs6861333	G	C	0.357853	-0.011	0.002	2.10E-09	30.25
rs357510	A	G	0.586481	-0.0123	0.002	2.23E-09	37.82
rs28361092	A	G	0.38171	-0.0133	0.002	3.42E-09	44.22
rs1906252	A	C	0.50497	0.00972	0.002	2.05E-08	23.62

rs756747	G	T	0.548708	0.0123	0.002	4.56E-11	37.82
rs322773	G	A	0.552684	0.00986	0.002	1.48E-08	24.30
rs6962879	G	C	0.632207	0.00993	0.002	1.89E-08	24.65
rs2533133	A	G	0.471173	0.0148	0.002	1.02E-12	54.76
rs11238438	C	G	0.424453	0.00958	0.002	3.73E-08	22.94
rs12698893	A	G	0.180915	-0.0153	0.002	1.04E-11	58.52
rs34121855	G	T	0.191849	0.0135	0.002	9.92E-09	45.56
rs641815	G	A	0.0616302	-0.0194	0.003	2.30E-08	41.82
rs800578	C	T	0.800199	0.0114	0.002	4.51E-08	32.49
rs28601761	G	C	0.409543	0.0112	0.002	1.57E-10	31.36
rs10105127	C	T	0.666004	0.0103	0.002	1.72E-08	26.52
rs1484559	C	T	0.782306	0.012	0.002	1.66E-08	36.00
rs55932213	G	A	0.701789	0.0127	0.002	1.61E-10	40.32
rs4743005	A	G	0.191849	-0.014	0.002	5.57E-10	49.00
rs41297207	T	C	0.0914513	-0.0171	0.003	1.16E-08	32.49

SNP: single nucleotide polymorphism; EA: effect allele; NEA: baseline allele; EAF: effect allele frequency; SE: standard error; P : P-value in GWAS summary dataset, all statistical tests were two-sided, $P < 5 \times 10^{-8}$ was considered genome-wide significant; F: F-statistic, the intensity of the genetic tool is assessed using the F-statistic, with a value exceeding 10 indicating satisfactory tool strength.

The genetic risk of the “alcohol consumption (drink per week)”is defined at the time of the interview or questionnaires, measured by two questions[1]:
a. In the past week, how many alcoholic beverages did you have?
b. Thinking about the pst year, on the average how many drinks did you have each week?
This phenotype capture alcohol use that is in the normal, or anyway nonpathological, range, that is important for understanding the biology of habitual alcohol use[2].

Table S4. Single nucleotide polymorphism loci used as instrumental variables for problematic alcohol use.

SNP	EA	NEA	EAF	Beta	SE	P	F
rs6421482	A	G	0.436	-0.016437	0.002603	2.71E-10	39.87
rs61767420	A	G	0.4	0.015317	0.002681	1.11E-08	32.64
rs1260326	T	C	0.403	-0.024563	0.002642	1.44E-20	86.44
rs494904	T	C	0.596	-0.021371	0.002696	2.25E-15	62.84
rs1402398	A	G	0.627	0.019088	0.002689	1.26E-12	50.39
rs9679319	T	G	0.48	-0.015647	0.002604	1.87E-09	36.11
rs13382553	A	G	0.766	-0.018415	0.003069	1.97E-09	36.00
rs2673136	A	G	0.639	-0.015802	0.002691	4.30E-09	34.48
rs62250713	A	G	0.368	0.016267	0.002689	1.45E-09	36.60
rs13129401	A	G	0.453	-0.023316	0.002618	5.29E-19	79.32
rs2602856	A	C	0.661	-0.015678	0.002732	9.54E-09	32.93
rs13125415	A	G	0.585	-0.024219	0.002669	1.15E-19	82.34
rs13135092	A	G	0.919	0.056302	0.004823	1.74E-31	136.27
rs2533200	C	G	0.516	-0.015305	0.002718	1.79E-08	31.71
rs2582405	T	C	0.237	0.017499	0.003043	8.89E-09	33.07
rs7900002	T	G	0.601	-0.014549	0.002644	3.74E-08	30.28
rs56722963	T	C	0.255	-0.019028	0.002985	1.84E-10	40.63
rs576859	A	C	0.327	0.020327	0.003585	1.43E-08	32.15
rs6589386	T	C	0.432	-0.019549	0.002603	5.90E-14	56.40
rs10790456	A	G	0.78	0.023175	0.004123	1.90E-08	31.59
rs12296477	C	G	0.547	0.014307	0.002609	4.16E-08	30.07
rs61974485	T	C	0.265	0.016631	0.00302	3.65E-08	30.33
rs8008020	T	C	0.418	0.015959	0.002633	1.35E-09	36.74

rs72768626	A	G	0.945	0.03219	0.005757	2.25E-08	31.26
rs9937709	A	G	0.585	0.017382	0.002633	4.07E-11	43.58
rs492602	A	G	0.508	-0.015998	0.002604	8.07E-10	37.74

SNP: single nucleotide polymorphism; EA: effect allele; NEA: baseline allele; EAF: effect allele frequency; SE: standard error; P : P-value in GWAS summary dataset, all statistical tests were two-sided. $P < 5 \times 10^{-8}$ was considered genome-wide significant; F: F-statistic, the intensity of the genetic tool is assessed using the F-statistic, with a value exceeding 10 indicating satisfactory tool strength.

The genetic risk of the "problematic alcohol use" is defined at the combination of three parts[2]:
a. Alcohol use disorder (AUD) diagnosed through ICD-10/9;
b. lifetime DSM-IV diagnosis of alcohol dependence (AD);
c. AUDIT-P scores was assessed by 7 questions: 1). Frequency of inability to cease drinking; 2). Frequency of failure to fulfil normal expectations due to drinking alcohol; 3). Frequency of needing a morning drink of alcohol after a heavy drinking session; 4). Frequency of feeling guilt or remorse after drinking alcohol; 5). Frequency of memory loss due to drinking alcohol; 6). Been injured or injured someone else through drinking alcohol; 7). Had a relative, friend, or health worker who was concerned about or suggested a reduction in alcohol consumption.
"Problematic alcohol use" captures pathological alcohol use: physiological dependence and/or significant psychological, social or medical consequences.

Table S5. Characterization of outcome disease data.

Diseases	Data sources	Cases	Controls	ICD-10
**Mental and behavioral disorders**				
Depression	FinnGen	43280	329192	F32, F33
ADHD	FinnGen	462	371117	F90
Insomnia	FinnGen	4214	3514	F51.0, G47.0
**Diseases of the nervous system**				
Epilepsy	FinnGen	11740	287837	G40
Alzheimer's Disease	FinnGen	6489	170429	F00.1*, F00.10*, F00.10*G30.1, G30.1, G30.1+F00.10
Parkinson's Disease	FinnGen	4235	373042	G20
Multiple Sclerosis	FinnGen	2182	373987	G35
ALS	GWAS Meta-anlysis	22040	62644	G12.201
Migraine	FinnGen	18477	15905	G43
Normal-pressure hydrocephalus	FinnGen	767	375610	G91.2
**Digestive system diseases**				
Cholangitis	FinnGen	1715	330903	K83.0
Cholelithiasis	FinnGen	37041	330903	K80
Cholecystitis	FinnGen	4299	330903	K81
NAFLD	FinnGen	2275	375002	K76.0
Alcoholic Liver Disease	FinnGen	2761	366450	K70

Cirrhosis	FinnGen	3970	373307	K70,K74.0, K74.1,K74.2, K74.6,K74.6,I85
GERD	FinnGen	26184	320387	K21
Gastric Ulcer	FinnGen	5935	320387	K25
Duodenal Ulcer	FinnGen	3520	320387	K26
Acute Gastritis	FinnGen	2370	320387	K29.0, K29.1
Chronic Gastritis	FinnGen	9570	320387	K29.3, K29.4, K29.5
Acute Pancreatitis	FinnGen	6223	330903	K85
Chronic Pancreatitis	FinnGen	3320	330903	K86.00, K86.01, K86.08, K86.1
Acute Appendicitis	FinnGen	28745	346283	K35
Diverticular Disease	FinnGen	30649	301931	K57
Crohn's Disease	FinnGen	1665	375445	K50
Ulcerative Colitis	FinnGen	5034	371530	K51
Noninfective enteritis and colitis	FinnGen	7988	359927	K52
IBS	FinnGen	9323	301931	K58
Chronic Periodontitis	FinnGen	4434	259234	K05.30, K05.31
**Circulatory system disease**				
Heart failure	FinnGen	27304	349973	I11.0, I13.0, I13.2, I50
Peripheral vascular disease	FinnGen	2230	349539	I73
Coronary artery atherosclerosis	FinnGen	47550	313400	I24, I25, T82.2, Z95.1
Stroke	FinnGen	25398	339920	I61, I63, I64

Myocardial infarction	FinnGen	24185	313400	I21, I22
Atrial fibrillation	FinnGen	45766	191924	I48
Hypertension	FinnGen	111581	265626	I10-I15, I67.4
Venous thromboembolism	FinnGen	19372	357905	I26, I80, O87.1, O88.2
Aortic aneurysm	FinnGen	7395	349539	I71.1, I71.2, I71.3, I71.4, I71.5, I71.6, I71.8, I71.9
Varicose veins	FinnGen	29539	324121	I83
**Neoplasms**				
Colorectal cancer	FinnGen	6509	287137	C18, C19&, C20
Esophageal cancer	FinnGen	566	287137	C15
Gastric cancer	FinnGen	1307	287137	C16
Hepatocellular carcinoma	FinnGen	453	287137	C22.0&
Pancreatic cancer	FinnGen	1416	287137	C25
Breast cancer	FinnGen	15680	167189	C50
Ovarian cancer	FinnGen	1025	167189	C56
Endometrial cancer	FinnGen	1967	1677	C54
Cervical cancer	FinnGen	369	167189	C53
Prostate cancer	FinnGen	13216	119948	C61
Bladder cancer	FinnGen	2053	287137	C67
Kidney cancer	FinnGen	2223	287137	C64
Brain cancer	FinnGen	764	287137	C71

Head and Neck cancer	**FinnGen**	**2131**	**287137**	**C00&,C01&, C02, C03, C04, C05, C06, C07, C08,C09, C10,C11, C12&, C13, C14,C32**
Malignant melanoma	**FinnGen**	**3960**	**286874**	**C51.03&, C51.13&, C51.23&, C51.82&, C52.93&, C69.02&, C69.82&, C69.92&, D03.9&**
Non-Hodgkin lymphoma	**FinnGen**	**928**	**287137**	**C82, C83, C84, C85**
Multiple myeloma	**FinnGen**	**585**	**287129**	**C90.0**
**Other diseases**				
Spontaneous abortion	**FinnGen**	**16906**	**149622**	**O03**
Ectopic pregnancy	**FinnGen**	**5648**	**149622**	**O00**
Puerperal sepsis	**FinnGen**	**3940**	**202267**	**O85**
Hyperplasia of prostate	**FinnGen**	**30066**	**119297**	**N40**
Rheumatoid arthritis	**FinnGen**	**9243**	**368029**	**M05**
Low back pain	**FinnGen**	**2439**	**460571**	**M54.5**
Osteoporosis	**FinnGen**	**7300**	**358014**	**M80, M81, M82***
Lupus erythematosus	**FinnGen**	**652**	**353088**	**L93#**
Psoriasis	**FinnGen**	**9267**	**364071**	**L40**

Asthma	FinnGen	42163	202399	J45, J46
Pneumonia	FinnGen	58174	319103	J12,J171,<br>J100,J110,<br>B012,B068,<br>B250,J13,<br>J14,J15,<br>J17.0*, J17.0*A01.0,<br>J17.0*A02.2,<br>J17.0*A21.2,<br>J17.0*A22.1,<br>J17.0*A37.9,<br>J17.0*A42.0,<br>J17.0*A43.0,<br>J17.0*A54.8,<br>J16,J17[2-8],J18
COVID-19（severe）	HGI 7	18152	1145549	U071
URIT	FinnGen	69111	308166	J00-J06
Chronic rhinitis, nasopharyngitis and pharyngitis	FinnGen	10868	283342	J31
Senile cataract	FinnGen	59522	312864	H25
Glaucoma	FinnGen	18902	358375	H40-H42
Age-related macular degeneration	FinnGen	8913	348936	H35.31，H35.32，<br>H35.33
Diabetic retinopathy	FinnGen	10413	308633	E11.3+,<br>E11.3+H28.0,<br>E11.3+H36.09

ADHD: Attention Deficit Hyperactivity Disorder syndrome; ALS: Amyotrophic lateral sclerosis; NAFLD: Nonalcoholic Fatty Liver Disease; GERD: Gastroesophageal reflux; IBS: irritable bowel syndrome; URIT: Acute upper respiratory tract infection; FinnGen: FinnGen database(https://www.finngen.fi/en); HGI 7: version 7 data published by the COVID-19 Host Genetics Program (https://www.covid19hg.org/results/r7/); ICD-10: International Classification of Diseases, Tenth Revision.

Table S6. Mendelian randomization studies included in the meta-analyses of genetic liability to alcohol consumption in relation to Circulatory system diseases, Digestive system diseases, Nervous system diseases and mental and behavioral disorders, Neoplasms, and Other diseases.

Diseases	Data sources	Cases	Controls	NIVs	MR-PRESSO			IVW		Weight Median		MR-Egger			Author, years
					P-gt	Outliers	P-Q	OR(95%CI)	P	OR(95%CI)	P	OR(95%CI)	P	P intercept	
**Circulatory system disease**															
Heart failure	HFMETTC	47309	930014	91	NA	NA	NA	1.11（0.85,1.46）	0.302	1.28（0.89，1.86）	0.081	1.30（0.76,2.23）	0.211	NA	Oort et al; 2020
	UKB	6712	360874	NA	NA	NA	NA	1.00（0.68,1.47）	0.996	NA	NA	NA	NA	NA	Larsson et al; 2020
	FinnGen	27304	349973	96	0.006	NA	0.005	1.06（0.87,1.29）	0.564	1.21（0.93,1.56）	0.154	1.36（0.81,2.30）	0.246	0.307	De novo MR; 2023
	Meta-anlysis	81325	1640861					1.07（0.92,1.23）	0.398	NA	NA	NA	NA		
Peripheral vascular disease	UKB	3415	364171	NA	NA	NA	NA	3.05（1.92,4.85）	2.30E-06	NA	NA	NA	NA	NA	Larsson et al; 2020
	MVP	31307	211753	84	NA	NA	NA	1.12（0.84,1.49）	0.420	NA	NA	NA	NA	NA	Hoek et al; 2022

	van Zuydam et al	12086	499548	90	NA	NA	NA	1.87 (1.27,2.75)	0.002	NA	NA	NA	NA	NA	Hoek et al; 2022
	FinnGen	2230	349539	96	0.526	NA	0.515	0.99 (0.59,1.66)	0.965	0.84 (0.37,1.90)	0.669	0.56 (0.14,2.27)	0.419	0.394	De novo MR; 2023
	Meta-anlysis	49038	1425011					1.58 (0.97,2.58)	0.069	NA	NA	NA	NA	NA	
Coronary artery atherosclerosis	GWAS Meta-anlysis	122733	424528	71	NA	NA	<0.001	1.13 (0.87,1.48)	0.368	1.69 (1.05,2.73)	0.036	1.61 (1.28,2.04)	5.88E-05	0.054	Rosoff et al; 2020
	FinnGen	47550	313400	94	<0.001	2	<0.001	1.07 (0.87,1.32)	0.507	1.09 (0.86,1.39)	0.457	1.14 (0.65,2.00)	0.639	0.809	De novo MR; 2023
	Meta-anlysis	170283	737928					1.09 (0.93,1.29)	0.290	1.29 (0.85,1.96)	0.232	1.50 (1.13,1.97)	0.005		
Stroke	GWAS meta-anlysis	76814	812384	94	NA	NA	NA	1.27 (1.12,1.45)	2.87E-04	1.30 (1.07,1.58)	0.008	1.21 (0.97,1.49)	0.085	NA	Larsson et al; 2020
	FinnGen	25398	339920	95	<0.001	1	0.007	1.08 (0.89,1.31)	0.454	1.09 (0.83,1.44)	0.526	0.92 (0.55,1.56)	0.767	0.536	De novo MR; 2023

	Meta-anlysis	102212	1152304					1.19 (1.02,1.39)	0.028	1.22 (1.04,1.44)	0.015	1.16 (0.95,1.42)	0.136		
Myocardial infarction	CARDIoGRAMplusC4D	43676	128199	71	NA	NA	0.008	1.23 (0.99,1.53)	0.066	1.38 (1.00,1.93)	0.058	1.23 (0.94,1.60)	0.127	0.350	Rosoff et al; 2020
	FinnGen	24185	313400	95	<0.001	1	<0.001	1.11 (0.88,1.41)	0.376	1.24 (0.91,1.70)	0.173	1.52 (0.81,2.87)	0.194	0.296	De novo MR; 2023
	Meta-anlysis	67861	441599					1.17 (1.00,1.38)	0.050	1.30 (1.04,1.64)	0.021	1.27 (0.99,1.62)	0.056		
Atrial fibrillation	GWAS Meta-anlysis	60620	970216	42	0.068	1	NA	1.00 (0.77,1.32)	0.979	1.05 (0.75,1.48)	0.790	1.08 (0.36,3.26)	0.892	0.894	Jiang et al; 2020
	AFGen	65446	522744	94	NA	NA	NA	1.17 (1.00,1.37)	0.050	1.17 (0.93,1.46)	0.186	1.24 (0.94,1.62)	0.124	NA	Larsson et al;
	FinnGen	45766	191924	94	<0.001	2	<0.001	1.10 (0.88,1.38)	0.380	1.25 (0.93,1.66)	0.134	1.39 (0.77,2.53)	0.276	0.411	De novo MR; 2023
	Meta-anlysis	171832	1684884					1.12 (0.99,1.26)	0.061	1.17 (1.00,1.36)	0.057	1.26 (0.99,1.60)	0.065		

Hypertension	MRC-IEU	124227	337653	71	NA	NA	<0.001	1.05 (1.00,1.10)	0.033	1.04 (1.01,1.08)	0.017	1.14 (1.06,1.23)	0.001	0.011	Rosoff et al; 2020
	FinnGen	111581	265626	91	<0.001	5	<0.001	1.03 (0.86,1.22)	0.780	1.25 (1.05,1.49)	0.011	1.80 (1.03,3.14)	0.040	0.039	De novo MR; 2023
	Meta-anlysis	235808	603279					1.05 (1.00,1.10)	0.043	1.12 (0.94,1.33)	0.218	1.15 (1.07,1.24)	2.000E-04		
Venous thromboembolism	UKB	14097	353489	94	NA	2	NA	1.04 (0.77,1.39)	0.810	0.90 (0.59,1.38)	0.619	0.96 (0.55,1.68)	0.879	NA	Larsson et al; 2020
	FinnGen	19372	357905	96	0.062	NA	0.074	1.04 (0.85,1.27)	0.693	0.92 (0.69,1.23)	0.583	1.09 (0.63,1.86)	0.761	0.865	De novo MR; 2023
	Meta-anlysis	33469	711394					1.04 (0.88,1.23)	0.643	0.91 (0.72,1.16)	0.459	1.02 (0.69,1.51)	0.901		
Aortic aneurysm	UKB	1374	400595	68	NA	0	NA	1.96 (0.84,4.56)	0.121	8.12 (1.70,38.86)	0.011	6.93 (1.87,25.60)	0.001	NA	Zhou et al; 2022
	FinnGen	7395	349539	96	0.022	NA	0.020	1.06 (0.76,1.48)	0.735	0.78 (0.49,1.24)	0.289	0.60 (0.24,1.47)	0.266	0.183	De novo MR; 2023

	Meta-anlysis	8769	750134					1.27 (0.73,2.19)	0.395	2.22 (0.23,21.80)	0.493	1.94 (0.18,21.35)	0.586		
Varicose veins	UKB	6958	330241	84	NA	2	NA	1.30 (0.89,1.91)	0.178	1.37 (0.82,2.30)	0.235	1.83 (0.87,3.87)	0.118	0.303	Yuan et al; 2021
	FinnGen	29539	324121	95	<0.001	1	<0.001	1.17 (0.94,1.46)	0.152	1.27 (0.98,1.64)	0.066	1.11 (0.61,2.01)	0.731	0.841	De novo MR; 2023
	Meta-anlysis	36497	654362					1.20 (0.99,1.45)	0.060	1.29 (1.02,1.62)	0.031	1.35 (0.84,2.18)	0.217		
Digestive system diseases															
Cholangitis	UKB(lee)	477	391307	80	NA	NA	0.437	1.20 (0.33, 4.33)	0.779	9.52 (1.37, 66.37)	0.023	5.87 (0.51, 68.21)	0.161	0.142	Yuan et al;2023
	FinnGen	1715	330903	96	0.105	NA	0.105	0.81 (0.43,1.53)	0.518	1.39 (0.56,3.47)	0.478	0.32 (0.06,1.77)	0.196	0.256	De novo MR; 2023
	Meta-anlysis	2192	722210					0.87 (0.50,1.55)	0.645	2.98 (0.47,18.88)	0.246	1.19 (0.07,20.35)	0.904		
Cholelithiasis	UKB(neale)	10520	350674	82	NA	NA	NA	1.30 (0.82,2.06)	0.267	1.00 (0.60,1.65)	0.906	1.09 (0.45,2.68)	0.454	0.697	Yuan et al; 2022

	FinnGen	37041	330903	89	<0.001	7	<0.001	0.80 (0.58,1.11)	0.181	0.98 (0.76,1.26)	0.868	0.53 (0.18,1.56)	0.255	0.438	De novo MR; 2023
	Meta-anlysis	47561	681577					0.99 (0.62,1.59)	0.965	0.98 (0.78,1.23)	0.889	0.81 (0.41,1.63)	0.558		
Cholecystitis	UKB(lee)	13777	391307	80	NA	NA	0.319	0.83 (0.48, 1.44)	0.502	1.71 (0.69, 4.25)	0.249	1.20 (0.41, 3.52)	0.737	0.428	Yuan et al; 2023
	FinnGen	4299	330903	96	0.280	NA	0.284	1.26 (0.85,1.86)	0.242	1.04 (0.59,1.83)	0.902	0.85 (0.30,2.40)	0.756	0.423	De novo MR; 2023
	Meta-anlysis	18076	722210					1.07 (0.72,1.60)	0.736	1.20 (0.74,1.93)	0.467	1.00 (0.48,2.12)	0.991		
NAFLD	UKB(lee)	1664	400055	80	NA	3	<0.001	1.17 (0.49, 2.80)	0.727	3.43 (1.19, 9.88)	0.022	1.44 (0.26, 7.85)	0.678	0.782	Yuan et al;2023
	GWAS Meta-anlysis	8434	770180	74	0.060	4	0.329	0.72 (0.41,1.25)	0.241	0.98 (0.52,1.84)	0.954	0.35 (0.08,1.62)	0.184	NA	Yuan et al; 2022
	Anstee et al	1483	17781	84	0.102	3	0.719	0.41 (0.18,0.95)	0.036	0.88 (0.36,2.16)	0.779	0.27 (0.02,3.34)	0.308	NA	Yuan et al; 2022

	FinnGen	2275	375002	95	<0.001	1	<0.001	0.96 (0.49,1.89)	0.913	1.36 (0.59,3.13)	0.467	0.75 (0.12,4.58)	0.755	0.770	De novo MR; 2023
	Meta-anlysis	13856	1563018					0.76 (0.54,1.08)	0.127	1.29 (0.79,2.09)	0.311	0.62 (0.26,1.50)	0.292		
Alcoholic Liver Disease	FinnGen	2761	366450	96	0.071	NA	0.063	3.67 (2.18,6.19)	9.78E-07	4.77 (2.26,10.08)	4.08E-05	9.36 (2.11,41.41)	4.05E-03	0.192	De novo MR; 2023
Cirrhosis	UKB(lee)	2895	400055	80	NA	NA	0.081	1.16 (0.06, 20.81)	0.919	2.90 (0.03, 332.48)	0.660	1.13 (0.00, 314.58)	0.966	0.992	Yuan et al; 2023
	FinnGen	3970	373307	95	<0.001	1	<0.001	2.45 (1.50,3.99)	3.47E-04	3.14 (1.67,5.92)	3.99E-04	2.45 (0.66,9.12)	0.186	0.999	De novo MR; 2023
	Meta-anlysis	6865	773362					2.40 (1.48,3.89)	4.00E-04	3.14 (1.68,5.87)	4.00E-04	2.45 (0.66,9.11)	0.181		
GERD	UKB(lee)	14223	369275	80	NA	NA	0.002	0.85 (0.63,1.14)	0.274	0.78 (0.49,1.23)	0.282	0.87 (0.49,1.54)	0.630	0.925	Yuan et al; 2023
	GWAS meta-anlysis	80265	305011	71	NA	NA	NA	1.00 (0.81,1.23)	0.987	0.98 (0.78,1.22)	0.839	0.92 (0.61,1.37)	0.676	0.618	Yuan et al; 2022
	FinnGen	26184	320387	96	0.006	NA	0.006	1.02 (0.85,1.23)	0.823	1.12 (0.87,1.45)	0.376	1.29 (0.78,2.13)	0.315	0.321	De novo MR; 2023

	Meta-anlysis	120672	994673					1.02（0.89,1.17）	0.784	1.00（0.86,1.18）	0.961	1.01（0.76,1.33）	0.968		
Gastric Ulcer	UKB(lee)	4109	401525	80	NA	NA	0.385	1.04（0.67,1.63）	0.858	1.34（0.62,2.89）	0.456	1.11（0.47,2.65）	0.813	0.866	Yuan et al; 2023
	FinnGen	5935	320387	96	0.395	NA	0.401	1.38（1.00,1.91）	0.053	1.93（1.18,3.16）	0.009	1.32（0.55,3.17）	0.531	0.924	De novo MR; 2023
	Meta-anlysis	10044	721912					1.25（0.96,1.62）	0.097	1.73（1.15,2.63）	0.009	1.21（0.66,2.24）	0.541		
Duodenal Ulcer	UKB(lee)	3002	401525	80	NA	NA	0.167	1.65（0.95,2.86）	0.077	0.92（0.39,2.16）	0.843	0.96（0.33,2.82）	0.947	0.258	Yuan et al; 2023
	FinnGen	3520	320387	96	0.103	NA	0.105	2.01（1.28,3.15）	0.002	2.44（1.25,4.75）	0.009	2.59（0.77,8.66）	0.126	0.657	De novo MR; 2023
	Meta-anlysis	6522	721912					1.86（1.31,2.63）	0.001	1.56（0.60,4.04）	0.363	1.51（0.57,3.99）	0.402		
Acute Gastritis	UKB(lee)	1184	378124	80	NA	NA	0.650	1.29（0.59,2.83）	0.522	0.65（0.16,2.66）	0.553	0.90（0.19,4.30）	0.892	0.595	Yuan et al; 2023

	FinnGen	2370	320387	96	0.068	NA	0.070	0.95 (0.54,1.64)	0.843	1.10 (0.48,2.50)	0.823	1.52 (0.35,6.72)	0.580	0.499	De novo MR; 2023
	Meta-anlysis	3554	698511					1.05 (0.67,1.66)	0.826	0.96 (0.47,1.96)	0.913	1.19 (0.41,3.47)	0.755		
Chronic Gastritis	UKB(lee)	370	378124	80	NA	NA	0.425	6.03 (1.38,26.24)	0.017	1.55 (0.15,15.59)	0.709	1.41 (0.08,25.04)	0.816	0.253	Yuan et al; 2023
	FinnGen	9570	320387	96	0.423	NA	0.414	1.19 (0.92,1.54)	0.180	1.47 (0.99,2.18)	0.056	2.57 (1.30,5.06)	0.008	0.019	De novo MR; 2023
	Meta-anlysis	9940	698511					2.26 (0.48,10.72)	0.304	1.47 (1.00,2.17)	0.051	2.49 (1.29,4.82)	0.007		
Acute Pancreatitis	UKB(lee)	1986	406271	80	NA	NA	0.108	0.72 (0.36,1.44)	0.355	0.42 (0.14,1.26)	0.122	0.76 (0.20,2.88)	0.684	0.938	Yuan et al; 2023
	FinnGen	6223	330903	96	0.006	NA	0.007	1.46 (1.01,2.11)	0.043	2.01 (1.23,3.29)	0.006	3.26 (1.21,8.76)	0.021	0.090	De novo MR; 2023
	Meta-anlysis	8209	737174					1.25 (0.90,1.73)	0.180	1.00 (0.22,4.58)	0.995	1.94 (0.88,4.30)	0.101		

Chronic Pancreatitis	UKB(lee)	514	406271	80	NA	NA	0.101	2.75（0.71,10.62)	0.141	3.60（0.33,38.88)	0.291	16.72（1.26,221.90)	0.036	0.114	Yuan et al; 2023
	FinnGen	3320	330903	96	0.058	NA	0.049	1.90（1.18,3.05)	0.008	2.45（1.27,4.74)	0.008	2.05（0.56,7.45)	0.279	0.901	De novo MR; 2023
	Meta-anlysis	3834	737174					1.98（1.26,3.10)	0.003	2.52（1.33,4.75)	0.004	3.12（0.98,9.93)	0.054		
Acute Appendicitis	UKB(lee)	2608	405552	80	NA	NA	0.498	0.95（0.55,1.65)	0.866	0.66（0.24,1.80)	0.421	0.57（0.20,1.64)	0.298	0.265	Yuan et al; 2023
	FinnGen	28745	346283	96	0.132	NA	0.150	0.97（0.83,1.14)	0.747	1.07（0.84,1.36)	0.575	0.53（0.36,0.80)	0.003	0.002	De novo MR; 2023
	Meta-anlysis	31353	751835					0.97（0.83,1.13)	0.680	1.04（0.82,1.32)	0.728	0.53（0.37,0.78)	0.001		
Diverticular Disease	UKB(lee)	3195	334783	80	NA	1	<0.001	1.15（0.87,1.52)	0.333	1.09（0.78,1.51)	0.617	1.13（0.66,1.94)	0.664	0.941	Yuan et al; 2023
	FinnGen	30649	301931	95	<0.001	1	<0.001	0.90（0.73,1.10)	0.308	0.95（0.74,1.21)	0.670	0.87（0.50,1.51)	0.620	0.903	De novo MR; 2023

	Meta-anlysis	33844	636714					0.98 (0.83,1.15)	0.818	1.00 (0.82,1.22)	0.982	0.99 (0.68,1.46)	0.979		
Crohn's Disease	UKB(lee)	1743	334783	80	NA	NA	0.394	0.98 (0.50,1.93)	0.953	0.58 (0.18,1.86)	0.361	0.58 (0.16,2.15)	0.420	0.304	Yuan et al; 2023
	IIBDGC	5956	14927	82	NA	5	<0.001	0.66 (0.33,1.31)	0.231	0.98 (0.54,1.79)	0.948	0.82 (0.27,2.46)	0.720	0.623	Yuan et al; 2023
	GWAS Meta-anlysis	2194	25042	90	NA	NA	NA	1.73 (1.04,2.86)	0.030	1.36 (0.56,3.27)	0.500	1.03 (0.63,1.70)	0.910	0.510	Georgiou et al; 2021
	FinnGen	1665	375445	95	0.032	1	0.027	0.94 (0.47,1.90)	0.869	1.32 (0.47,3.73)	0.603	0.14 (0.01,1.31)	0.088	0.082	De novo MR; 2023
	Meta-anlysis	11558	750197					1.11 (0.81,1.52)	0.502	1.04 (0.68,1.57)	0.870	0.88 (0.58,1.35)	0.570		
Ulcerative Colitis	UKB(lee)	3195	334783	80	NA	NA	0.003	0.98 (0.53,1.79)	0.937	0.97 (0.39,2.40)	0.945	0.76 (0.23,2.48)	0.651	0.630	Yuan et al; 2023
	IIBDGC	5956	14927	82	NA	3	<0.001	0.99 (0.59,1.66)	0.960	0.99 (0.51,1.92)	0.983	1.02 (0.43,2.45)	0.961	0.922	Yuan et al; 2023

	GWAS Meta-anlysis	12366	25042	90	NA	NA	NA	1.57 (0.92,2.66)	0.100	1.44 (0.57,3.65)	0.440	0.97 (0.59,1.61)	0.830	0.830	Georgiou et al; 2021
	FinnGen	5034	371530	95	0.001	1	0.001	1.01 (0.65,1.56)	0.960	1.13 (0.64,2.00)	0.682	0.42 (0.13,1.39)	0.156	0.124	De novo MR; 2023
	Meta-anlysis	26551	746282					1.11 (0.86,1.43)	0.433	1.10 (0.77,1.58)	0.602	0.91 (0.68,1.23)	0.543		
Noninfective enteritis and colitis	FinnGen	7988	359927	96	0.295	NA	0.289	1.12 (0.84,1.48)	0.451	1.15 (0.75,1.74)	0.523	1.64 (0.76,3.54)	0.207	0.289	De novo MR; 2023
IBS	UKB(lee)	5548	334783	80	NA	NA	0.071	0.88 (0.57,1.34)	0.538	0.96 (0.49,1.90)	0.915	0.59 (0.26,1.34)	0.214	0.278	Yuan et al; 2023
	GERA	3117	53520	79	NA	NA	0.346	0.50 (0.28,0.90)	0.022	0.37 (0.15,0.94)	0.036	0.26 (0.05,1.38)	0.118	0.413	Yuan et al; 2023
	FinnGen	9323	301931		0.289	NA	0.309	0.90 (0.69,1.17)	0.415	1.09 (0.73,1.61)	0.679	1.15 (0.57,2.35)	0.695	0.455	De novo MR; 2023
	Meta-anlysis	17988	690234					0.83 (0.67,1.02)	0.081	0.93 (0.675,1.280)	0.652	0.77 (0.46,1.29)	0.320		

Chronic Periodontitis	GLIDE	17353	28210	33	0.034	NA	0.467	1.41 (1.04,1.90)	0.027	NA	NA	NA	NA	0.822	Baumeister et al; 2021
	FinnGen	4434	259234	96	0.029	NA	0.029	1.65 (1.09,2.51)	0.018	1.68 (0.92,3.07)	0.093	2.24 (0.73,6.87)	0.163	0.571	De novo MR; 2023
	Meta-anlysis	21787	287444					1.49 (1.17,1.90)	0.001	NA	NA	NA	NA		
Mental and behavioral disorder															
Depression	FinnGen	43280	329192	95	<0.001	1	<0.001	1.16 (0.95,1.40)	0.141	1.25 (0.99,1.57)	0.060	1.15 (0.68,1.94)	0.604	0.982	De novo MR; 2023
ADHD	FinnGen	462	371117	91	<0.001	5	<0.001	1.03 (0.86,1.22)	0.780	1.25 (1.05,1.49)	0.011	1.80 (1.03,3.14)	0.040	0.039	De novo MR; 2023
Insomnia	GWAS Meta-anlysis	NA	NA	78	NA	NA	NA	1.13 (0.87,1.45)	0.349	1.10 (0.75, 1.59)	0.624	1.12 (0.74,1.68)	0.579	NA	Pasman et al;2020
	FinnGen	4214	3514	96	0.825	NA	0.822	1.14 (0.78,1.66)	0.500	0.91 (0.52,1.60)	0.755	1.01 (0.36,2.82)	0.985	0.805	De novo MR; 2023
	Meta-anlysis	NA	NA					1.13 (0.92,1.40)	0.247	1.04 (0.76,1.42)	0.818	1.10 (0.75,1.62)	0.610		
Nervous system diseases															

Epilepsy	ILAE	15212	29677	62	NA	NA	NA	1.22 (1.02,1.45)	0.028	1.30 (0.99,1.70)	0.055	1.37 (0.83,2.28)	0.219	NA	Zhang et al;2022
	FinnGen	11740	287837	96	0.130	NA	0.128	1.15 (0.90.1.47)	0.265	1.27 (0.90,1.79)	0.172	1.22 (0.62,2.41)	0.558	0.847	De novo MR; 2023
	Meta-anlysis	26952	317514					1.20 (1.04,1.38)	0.014	1.29 (1.04,1.59)	0.019	1.31 (0.88,1.97)	0.186		
Alzheimer's Disease	Lambert et al	17008	37154	41	0.058	1	NA	0.96(0.71,1.22)	0.775	0.81(0.44,1.18)	0.263	0.81(0.38,1.24)	0.353	0.286	Andrews et al;2020
	FinnGen	6489	170429	95	0.006	1	0.004	0.96 (0.63,1.46)	0.847	1.23 (0.72,2.12)	0.444	1.19 (0.39,3.62)	0.764	0.687	De novo MR; 2023
	Meta-anlysis	23497	207583					0.96 (0.76,1.21)	0.725	0.98 (0.65,1.48)	0.935	0.88 (0.52,1.49)	0.636		
Parkinson's Disease	iPDGC	37688	981372	33	NA	NA	NA	0.79 (0.65,0.96)	0.021	0.75 (0.60,0.95)	0.017	0.73 (0.56,0.95)	0.019	NA	Domínguez-Baleón et al;2021
	Courage-PD	7369	7018	62	NA	NA	NA	0.68 (0.39,1.18)	0.170	0.86 (0.42,1.75)	0.670	0.70 (0.30,1.16)	0.400	0.940	Domenighetti et al; 2022

	FinnGen	4235	373042	95	0.343	NA	0.345	0.84 (0.57,1.24)	0.381	0.77 (0.42,1.39)	0.387	0.86 (0.29,2.53)	0.791	0.954	De novo MR; 2023
	Meta-anlysis	49292	1361432					0.79 (0.67,0.93)	0.005	0.76 (0.62,0.93)	0.009	0.73 (0.58,0.93)	0.011		
Multiple Sclerosis	IMSGC	47429	68374	67	NA	NA	NA	0.85 (0.50,1.42)	0.540	1.12 (0.60,2.13)	0.710	3.01 (0.62,14.71)	0.180	0.100	Jiang et al;2021
	FinnGen	2182	373987	96	<0.001	NA	0.001	0.37 (0.19,0.70)	0.002	0.46 (0.20,1.07)	0.072	0.80 (0.14,4.49)	0.796	0.345	De novo MR; 2023
	Meta-anlysis	49611	442361					0.57 (0.25,1.30)	0.182	0.75 (0.32,1.79)	0.516	1.63 (0.45,5.95)	0.460		
ALS	GWAS meta-anlysis	22040	62644	100	0.023	NA	0.016	0.93 (0.67,1.30)	0.691	0.91 (0.69,1.19)	0.490	0.93 (0.67,1.30)	0.691	0.871	De novo MR; 2023
Migraine	UKB	1072	360122	82	NA	NA	0.116	0.53 (0.20,1.41)	0.204	1.94 (0.42,8.88)	0.392	1.66 (0.25,11.01)	0.600	0.171	Yuan et al; 2022
	FinnGen	18477	15905	96	0.008	1	0.009	0.83 (0.67,1.04)	0.104	0.77 (0.57,1.04)	0.083	0.61 (0.34,1.11)	0.109	0.275	De novo MR; 2023

	Meta-anlysis	19549	376027					0.81（0.66,1.01）	0.057	0.80（0.59,1.07）	0.132	0.67（0.38,1.17）	0.160		
Normal-pressure hydrocephalus	FinnGen	767	375610	96	0.104	NA	0.107	1.13（0.43,2.96）	0.796	1.18（0.29,4.85）	0.815	1.01（0.07,13.80）	0.996	0.923	De novo MR; 2023
Neoplasms															
Colorectal cancer	Li et al	16871	26328	84	NA	NA	<0.001	1.79（1.23,2.61）	0.003	1.49（0.91,2.43）	0.112	2.03（0.93,4.43）	0.080	0.717	Zhou et al; 2022
	UKB(Larsson)	5486	NA	NA	0.001	NA	NA	1.31 (0.84,2.04)	0.235	0.89 (0.48,1.68)	0.729	1.10 (0.46,2.59)	0.833	0.638	Larsson et al; 2020
	FinnGen	6509	287137	95	0.003	1	0.003	1.01（0.70,1.47）	0.943	1.03（0.61,1.74）	0.922	1.29（0.47,3.51）	0.622	0.614	De novo MR; 2023
	Meta-anlysis	28866	NA					1.33（1.06,1.67）	0.013	1.15（0.84,1.57）	0.372	1.47（0.89,2.44）	0.129		
Esophageal cancer	UKB(lee)	720	393372	80	NA	NA	0.274	4.10 (1.39, 12.09)	0.011	8.93 (1.60, 50.01)	0.013	2.32 (0.29, 18.64)	0.431	0.532	Yuan et al; 2023
	FinnGen	566	287137	96	0.458	NA	0.455	1.58（0.57,4.42）	0.379	4.33（0.88,21.16）	0.070	7.49（0.49,115.74）	0.153	0.233	De novo MR; 2023

	Meta-anlysis	1286	NA					2.48（1.18,5.22）	0.017	6.04（1.88,19.43）	0.003	3.57（0.68,18.69）	0.132		
Gastric cancer	UKB(lee)	554	393372	80	NA	NA	0.682	2.08 (0.67, 6.45)	0.203	2.05 (0.24, 17.37)	0.512	1.76 (0.18, 17.10)	0.626	0.867	Yuan et al; 2023
	FinnGen	1307	287137	96	0.319	NA	0.314	1.83（0.91,3.67）	0.089	0.88（0.30,2.61）	0.815	2.44（0.38,15.77）	0.352	0.746	De novo MR; 2023
	Meta-anlysis	1861	NA					1.90（1.05,3.43）	0.035	1.05（0.40,2.74）	0.929	2.14（0.51,9.05）	0.300		
Hepatocellular carcinoma	UKB(lee)	196	393372	80	NA	NA	0.372	1.76 (0.38, 8.11)	0.468	1.81 (0.11, 30.86)	0.683	0.36 (0.02, 6.75)	0.495	0.218	Yuan et al; 2023
	FinnGen	453	287137	96	0.115	NA	0.113	2.47（0.72,8.52）	0.151	4.93（0.81,30.16）	0.088	2.01（0.07,54.65）	0.680	0.849	De novo MR; 2023
	Meta-anlysis	649	680509					2.16（0.83,5.65）	0.116	3.68（0.80,16.86）	0.093	0.76（0.08,6.79）	0.805		
Pancreatic cancer	UKB(Larsson)	1264	NA	NA	0.386	NA	NA	1.16（0.55,2.43）	0.703	0.61（0.18,2.02）	0.411	1.11（0.27,4.66）	0.884	0.951	Yuan et al; 2023
	FinnGen	1416	287137	96	0.685	NA	0.675	0.56（0.29,1.07）	0.078	0.64（0.24,1.73）	0.381	0.84（0.15,4.76）	0.841	0.624	De novo MR; 2023

	Meta-anlysis	2680	NA					0.77 (0.47,1.26)	0.294	0.63 (0.29,1.35)	0.233	0.99 (0.33,2.98)	0.988		
Breast cancer	BCAC	133384	113789	84	0.898	NA	<0.001	1.01 (0.84,1.23)	0.883	0.86 (0.71,1.04)	0.128	1.01 (0.72,1.41)	0.977	0.947	Zhou et al; 2022
	GWAS meta-anlysis	122977	105974	NA	0.000	1	NA	0.99 (0.83,1.18)	0.922	0.93 (0.76,1.14)	0.498	0.95 (0.70,1.28)	0.721	0.731	Larsson et al; 2020
	UKB	13666	NA	NA	0.290	NA	NA	1.10 (0.87,1.40)	0.423	1.42 (0.96,2.08)	0.078	1.42 (0.89,2.25)	0.138	0.213	Larsson et al; 2020
	FinnGen	15680	167189	96	0.001	NA	0.001	1.13 (0.87,1.46)	0.365	1.02 (0.72,1.45)	0.915	0.91 (0.45,1.83)	0.797	0.525	De novo MR; 2023
	Meta-anlysis	285707	NA					1.04 (0.94,1.15)	0.481	0.95 (0.84,1.07)	0.415	1.04 (0.85,1.26)	0.714		
Ovarian cancer	OCAC	25509	40941	NA	0.556	NA	NA	0.91 (0.72,1.15)	0.448	0.74 (0.51,1.08)	0.121	0.79 (0.53,1.18)	0.251	0.385	Larsson et al; 2020
	UKB	1520	NA	NA	0.309	NA	NA	1.23 (0.62,2.47)	0.554	0.54 (0.17,1.69)	0.289	0.52 (0.14,1.98)	0.339	0.139	Larsson et al; 2020

	FinnGen	1025	167189	95	0.007	2	0.005	0.35 (0.14,0.85)	0.021	0.24 (0.07,0.81)	0.021	0.04 (0.00,0.39)	0.006	0.044	De novo MR; 2023
	Meta-anlysis	28054	NA					0.89 (0.72,1.10)	0.277	0.66 (0.47,0.93)	0.017	0.76 (0.52,1.12)	0.166		
Endometrial cancer	UKB	1931	NA	NA	0.208	NA	NA	0.84 (0.45,1.58)	0.591	1.24 (0.40,3.79)	0.708	2.04 (0.61,6.83)	0.247	0.092	Larsson et al; 2020
	FinnGen	1967	1677	96	0.042	NA	0.043	0.85 (0.45,1.58)	0.601	1.42 (0.58,3.45)	0.439	1.66 (0.31,9.05)	0.558	0.402	De novo MR; 2023
	Meta-anlysis	3898	NA					0.85 (0.54,1.32)	0.457	1.35 (0.67,2.71)	0.403	1.90 (0.71,5.08)	0.199		
Cervical cancer	UKB	1928	NA	NA	0.809	NA	NA	1.33 (0.73,2.42)	0.351	1.26 (0.43,3.75)	0.672	0.77 (0.24,2.45)	0.658	0.280	Larsson et al; 2020
	FinnGen	369	167189	96	0.265	NA	0.263	1.19 (0.32,4.46)	0.796	1.89 (0.27,13.37)	0.522	0.79 (0.02,28.47)	0.898	0.811	De novo MR; 2023
	Meta-anlysis	2297	NA					1.30 (0.76,2.25)	0.339	1.30 (0.76,2.25)	0.339	1.39 (0.54,3.57)	0.499		

Prostate cancer	PRACTICAL	79148	NA	NA	<0.001	7	NA	0.96 (0.74,1.24)	0.753	1.23 (0.95,1.61)	0.118	1.15 (0.73,1.82)	0.550	0.349	Larsson et al; 2020
	UKB	7872	NA	NA	<0.001	1	NA	0.91 (0.62,1.34)	0.630	1.02 (0.57,1.82)	0.956	1.01 (0.48,2.10)	0.980	0.745	Larsson et al; 2020
	FinnGen	13216	119948	94	<0.001	2	<0.001	0.86 (0.63,1.17)	0.331	1.01 (0.68,1.49)	0.964	1.30 (0.58,2.90)	0.528	0.284	De novo MR; 2023
	Meta-anlysis	100236	NA					0.92 (0.77,1.09)	0.330	1.14 (0.93,1.40)	0.214	1.14 (0.81,1.62)	0.454		
Lung cancer	ILCCO	11348	NA	NA	0.191	NA	NA	1.94 (1.41,2.68)	4.68E-05	2.04 (1.22,3.40)	0.006	2.03 (1.20,3.44)	0.008	0.834	Larsson et al; 2020
	UKB	2838	NA	NA	0.079	NA	NA	1.12 (0.65,1.93)	0.686	0.90 (0.36,2.26)	0.828	0.75 (0.26,2.14)	0.589	0.381	Larsson et al; 2020
	Meta-anlysis	14186	NA					1.68 (1.28,2.22)	2.00E-04	1.68 (1.07,2.63)	0.023	1.66 (1.04,2.66)	0.034		
Bladder cancer	UKB	2883	417955	NA	0.608	NA	0.598	0.64 (0.38,1.07)	0.090	0.51 (0.22,1.15)	0.108	0.36 (0.12,1.04)	0.060	0.227	Xiong et al; 2022

	FinnGen	2053	287137	96	0.030	NA	0.029	0.96 (0.51,1.78)	0.885	1.01 (0.41,2.45)	0.987	0.98 (0.19,5.19)	0.983	0.972	De novo MR; 2023
	Meta-anlysis	4936	705092					0.75 (0.51,1.12)	0.167	0.70 (0.36,1.37)	0.300	0.49 (0.20,1.20)	0.117		
Oral and Oropharyngeal cancer	GAME-OA	6034	6585	60	NA	NA	NA	9.96 (5.33,18.6)	5.64E-13	30.0 (12.6,71.5)	1.47E-14	36.7 (14.1,95.5)	6.08E-05	NA	Gormley et al; 2020
Kidney cancer	UKB	1310	NA	NA	0.506	NA	NA	0.90 (0.44,1.86)	0.786	0.65 (0.21,2.01)	0.460	0.40 (0.10,1.61)	0.200	0.181	Larsson et al; 2020
	FinnGen	2223	287137	96	0.246	NA	0.246	0.53 (0.31,0.91)	0.022	0.39 (0.17,0.88)	0.024	0.39 (0.09,1.67)	0.205	0.646	De novo MR; 2023
	Meta-anlysis	3533	NA					0.64 (0.42,0.99)	0.043	0.47 (0.24,0.90)	0.024	0.40 (0.14,1.08)	0.071		
Brain cancer	UKB	810	NA	NA	0.120	NA	NA	0.69 (0.26,1.87)	0.467	1.91 (0.40,9.14)	0.417	1.56 (0.23,10.57)	0.651	0.331	Larsson et al; 2020
	FinnGen	764	287137	96	0.573	NA	0.572	1.88 (0.78,4.53)	0.158	1.66 (0.43,6.35)	0.461	0.50 (0.05,5.26)	0.563	0.236	De novo MR; 2023

	Meta-anlysis	1574	NA					1.21（0.63,2.33)	0.576	1.76（0.64,4.89)	0.277	0.99（0.22,4.32)	0.985		
Head and Neck cancer	UKB	1615	NA	NA	0.024	NA	NA	1.75（0.83,3.72)	0.144	2.36（0.71,7.79)	0.160	2.23（0.51,9.68)	0.283	0.706	Larsson et al; 2020
	FinnGen	2131	287137	96	0.268	NA	0.257	1.91（1.10,3.32)	0.022	2.30（1.01,5.24)	0.048	2.72（0.62,12.05)	0.190	0.615	De novo MR; 2023
	Meta-anlysis	3746	NA					1.85（1.19,2.89)	0.007	2.32（1.18,4.57)	0.015	2.46（0.87,7.00)	0.091		
Malignant melanoma	UKB	4869	NA	NA	0.216	NA	NA	0.68（0.46,1.02)	0.059	0.60（0.32,1.32)	0.232	0.54（0.25,1.16)	0.115	0.483	Larsson et al; 2020
	FinnGen	3960	286874	70	0.082	NA	0.081	1.03（0.63,1.69)	0.913	0.91（0.44,1.90)	0.810	1.72（0.49,6.02)	0.397	0.381	De novo MR; 2023
	Meta-anlysis	8829	NA					0.81（0.54,1.22)	0.320	0.73（0.44,1.22)	0.234	0.86（0.28,2.63)	0.795		
Non-Hodgkin lymphoma	UKB	2296	NA	NA	0.154	NA	NA	0.66（0.37,1.18)	0.157	0.79（0.30,2.07)	0.626	0.88（0.28,2.69)	0.820	0.553	Larsson et al; 2020

	FinnGen	928	287137	96	0.516	NA	0.513	0.98 (0.44,2.18)	0.957	1.14 (0.33,3.97)	0.837	1.42 (0.17,11.95)	0.750	0.715	De novo MR; 2023
	Meta-anlysis	3224	NA					0.76 (0.47,1.21)	0.244	0.91 (0.42,1.94)	0.802	0.98 (0.36,2.65)	0.965		
Leukemia	UKB	1403	NA	NA	0.523	NA	NA	0.51 (0.25,1.02)	0.056	0.59 (0.15,2.29)	0.447	0.68 (0.18,2.59)	0.574	0.612	Larsson et al; 2020
Multiple myeloma	UKB	656	NA	NA	0.216	NA	NA	1.15 (0.41,3.21)	0.795	2.38 (0.40,14.37)	0.344	1.60 (0.22,11.68)	0.643	0.700	Larsson et al; 2020
	FinnGen	585	287129	96	0.487	NA	0.495	0.78 (0.29,2.14)	0.632	1.40 (0.31,6.32)	0.662	1.44 (0.10,21.40)	0.792	0.634	De novo MR; 2023
	Meta-anlysis	1241	NA					0.94 (0.46,1.93)	0.870	2.02 (0.64,6.39)	0.233	1.54 (0.31,7.61)	0.595		
Other diseases															
Pregnancy, childbirth and the puerperium															
Spontaneous abortion	UKB(neale)	60565	130687	84	0.342	NA	NA	1.09 (0.93,1.29)	0.300	1.04 (0.81,1.34)	0.756	0.86 (0.63,1.18)	0.350	0.090	Yuan et al; 2021
	FinnGen	16906	149622	96	0.550	NA	0.557	1.35 (1.11,1.64)	0.003	1.29 (0.95,1.75)	0.109	1.11 (0.65,1.89)	0.694	0.444	De novo MR; 2023

	Meta-anlysis	77471	280309					1.19（1.05,1.35）	0.006	1.13（0.93,1.38）	0.202	0.92（0.70,1.20）	0.537		
Ectopic pregnancy	GWAS Meta-anlysis	3556	327733	88	NA	NA	NA	1.49（0.52,4.23）	0.460	1.65（0.39,7.03）	0.500	1.08（0.03,35.44）	0.970	NA	Rogne et al; 2022
	FinnGen	5648	149622	96	0.009	NA	0.010	1.01（0.69,1.48）	0.968	1.17（0.70,1.97）	0.544	0.63（0.22,1.77）	0.379	0.335	De novo MR; 2023
	Meta-anlysis	9204	477355					1.06（0.74,1.51）	0.762	1.22（0.75,1.98）	0.430	0.66（0.24,1.79）	0.412		
Puerperal sepsis	FinnGen	3940	202267	96	0.990	NA	0.991	1.07（0.73,1.59）	0.723	1.07（0.59,1.93）	0.834	0.52（0.18,1.50）	0.230	0.153	De novo MR; 2023
Diseases of the genitourinary system															
Hyperplasia of prostate	UKB	14126	169762	38	0.417	NA	0.407	0.82（0.64,1.04）	0.101	NA	NA	NA	NA	0.670	Wang et al; 2022
	FinnGen	30066	119297	96	<0.001	NA	<0.001	0.79（0.63,0.98）	0.033	0.77（0.59,1.02）	0.066	0.80（0.45,1.45）	0.472	0.939	De novo MR; 2023
	Meta-anlysis	44192	289059					0.80（0.68,0.95）	0.009	NA	NA	NA	NA		

UTI	UKB	21958	464256	71	NA	NA	NA	1.01（0.80,1.27）	0.926	0.95（0.70,1.29）	0.729	1.54（0.70,3.40）	0.289	NA	Zhu et al; 2023
Diseases of the musculoskeletal system and connective tissue															
Rheumatoid arthritis	Okada et al;	19234	61565	68	NA	NA	NA	0.51（0.30,0.88）	0.020	0.88（0.42,1.85）	0.730	2.08（0.42,10.30）	0.370	0.070	Jiang et al; 2021
	FinnGen	9243	368029	96	0.124	NA	0.110	0.73（0.55,0.96）	0.025	0.76（0.50,1.14）	0.185	0.41（0.19,0.88）	0.025	0.123	De novo MR; 2023
	Meta-anlysis	28477	429594					0.68（0.53,0.87）	0.002	0.79（0.55,1.13）	0.219	0.56（0.28,1.11）	0.095		
Low back pain	FinnGen	2439	460571	95	<0.001	1	<0.001	1.04（0.85,1.28）	0.711	1.13（0.88,1.46）	0.336	1.03（0.59,1.81）	0.915	0.976	De novo MR; 2023
	MRC-IEU	29329	270964	92	0.921	NA	0.921	1.00（1.00,1.00）	0.450	1.00（1.00,1.00）	0.732	1.00（0.99,1.01）	0.392	0.503	De novo MR; 2023
	Meta-anlysis	31768	731535					1.0010（0.9984,1.0036）	0.448	1.0007（0.9969,1.0045）	0.721	1.0040（0.9950,1.0130）	0.389		
Osteoporosis	FinnGen	7300	358014	94	0.006	2	0.006	1.21（0.85,1.72）	0.280	1.22（0.77,1.94）	0.392	1.42（0.53,3.78）	0.487	0.739	De novo MR; 2023
Diseases of the skin and subcutaneous tissue															

Lupus erythematosus	GWAS meta-anlysis	5201	9066	60	NA	NA	NA	1.24 (0.49,3.15)	0.660	0.63 (0.20,2.02)	0.440	4.23 (0.31,57.18)	0.280	0.320	Jiang et al; 2021
	FinnGen	652	353088	96	0.723	NA	0.714	1.04 (0.40,2.69)	0.942	0.66 (0.16,2.78)	0.574	1.74 (0.13,22.79)	0.673	0.671	De novo MR; 2023
	Meta-anlysis	5853	362154					1.14 (0.58,2.21)	0.704	0.64 (0.26,1.58)	0.333	2.70 (0.43,16.93)	0.289		
Psoriasis	FinnGen	9267	364071	94	<0.001	2	<0.001	0.99 (0.71,1.40)	0.984	1.12 (0.71,1.77)	0.618	0.68 (0.22,2.05)	0.491	0.472	De novo MR; 2023
Diseases of the respiratory system															
Asthma	FinnGen	42163	202399	94	<0.001	2	<0.001	1.02 (0.85,1.23)	0.796	1.08 (0.86,1.36)	0.491	0.79 (0.48,1.30)	0.352	0.270	De novo MR; 2023
Pneumonia	UKB	22567	463917	71	NA	NA	NA	0.96 (0.76,1.22)	0.739	0.84 (0.62,1.15)	0.277	0.75 (0.36,1.69)	0.493	NA	Zhu et al; 2023
	FinnGen	58174	319103	96	0.007	NA	0.006	1.06 (0.93,1.21)	0.364	0.99 (0.82,1.20)	0.953	0.90 (0.63,1.29)	0.569	0.331	De novo MR; 2023
	Meta-anlysis	80741	783020					1.04 (0.92,1.16)	0.796	0.95 (0.80,1.11)	0.504	0.87 (0.63,1.21)	0.407		

COVID-19 (severe)	COVID-19 hg7	18152	1145549	106	0.438	NA	0.486	1.28 (1.00,1.64)	0.049	1.65 (1.13,2.43)	0.010	1.70 (1.14,2.53)	0.010	0.078	De novo MR; 2023
URIT	UKB	2795	483689	71	NA	NA	NA	0.94 (0.54,1.63)	0.827	0.73 (0.32,1.65)	0.450	0.49 (0.07,3.26)	0.461	NA	Zhu et al; 2023
	FinnGen	69111	308166	95	0.023	1	0.129	0.98 (0.87,1.09)	0.667	1.05 (0.88,1.25)	0.575	1.15 (0.85,1.56)	0.377	0.262	De novo MR; 2023
	Meta-anlysis	71906	791855					0.98 (0.88,1.09)	0.698	1.03 (0.87,1.23)	0.707	1.13 (0.83,1.52)	0.437		
Chronic rhinitis, nasopharyngitis and pharyngitis	FinnGen	10868	283342	95	<0.001	1	<0.001	0.99 (0.73,1.34)	0.966	0.88 (0.60,1.31)	0.532	0.70 (0.31,1.58)	0.390	0.364	De novo MR; 2023
Systemic Inflammatory Response Syndrome															
Sepsis	UKB	11643	474841	71	NA	NA	NA	0.99 (0.75,1.32)	0.967	0.95 (0.63,1.41)	0.780	1.36 (0.52,3.55)	0.534	NA	Zhu et al; 2023
Diseases of the eye and adnexa															
Senile cataract	UKB	6332	354862	84	NA	NA	NA	1.18 (0.78,1.79)	0.430	1.05 (0.55,2.00)	0.892	0.76 (0.34,1.71)	0.513	0.219	Yuan et al; 2022

	FinnGen	59522	312864	96	<0.001	NA	<0.001	0.95 (0.80,1.12)	0.540	1.01 (0.81,1.25)	0.944	0.78 (0.49,1.24)	0.294	0.375	De novo MR; 2023
	Meta-anlysis	65854	667726					0.98 (0.84,1.14)	0.794	1.01 (0.83,1.25)	0.895	0.78 (0.52,1.16)	0.215		
Glaucoma	FinnGen	18902	358375	95	<0.001	2	<0.001	1.06 (0.83,1.36)	0.631	1.16 (0.84,1.60)	0.358	0.80 (0.41,1.56)	0.517	0.374	De novo MR; 2023
Age-related macular degeneration	IAMDGC	16144	17832	NA	<0.001	3	NA	1.57 (1.03,2.40)	0.040	2.04 (1.23,3.39)	0.006	2.01 (0.98,4.10)	0.060	0.400	Kuan et al; 2021
	FinnGen	8913	348936	95	0.016	1	0.012	0.94 (0.68,1.30)	0.719	0.93 (0.60,1.42)	0.925	0.77 (0.33,1.82)	0.560	0.628	De novo MR; 2023
	Meta-anlysis	25057	366768					1.19 (0.72,1.97)	0.492	1.36 (0.63,2.94)	0.432	1.28 (0.50,3.28)	0.605		
Diabetic retinopathy	FinnGen	10413	308633	94	<0.001	2	<0.001	0.99 (0.72,1.36)	0.945	1.19 (0.79,1.79)	0.400	2.85 (1.02,7.93)	0.048	0.036	De novo MR; 2023

ADHD: Attention Deficit Hyperactivity Disorder syndrome; ALS: Amyotrophic lateral sclerosis; NAFLD: Nonalcoholic Fatty Liver Disease; GERD: Gastroesophageal reflux; IBS: irritable bowel syndrome ; URIT: Acute upper respiratory tract infection; ICD-10: International Classification of Diseases, Tenth Revision; NIVs: the number of Single Nucleotide Polymorphisms; P-gt: P-value tests for MR-PRESSO global pleiotropy; IVW: Multiplicative random-effects inverse-variance weighted; P-Q: p-value tests for the heterogeneity of Cochran's Q; OR: Odds ratio; CI: confidence interval; P-intercept: p-value tests for the intercept pleiotropy in MR-

Egger; All statistical tests were two-sided. $P < 0.05$ was considered significant; ILAE: international league against epilepsy; iPDGC: international parkinson's disease genomics consortium; Courage-PD: Courage parkinson's disease; IMSGC: international multiple sclerosis genetics consortium; IIBDGC: inflammatory bowel disease genetics consortium; GERA: genetic epidemiology research on aging; GLIDE: gene lifestyle interactions in dental endpoints; MRC-IEU: medical research council integrative epidemiology unit; IAMDGC: international age-related macular degeneration genomics consortium; HFMETTC :heart failure molecular epidemiology for therapeutic targets consortium; MVP: million veteran program; CARDIoGRAMplusC4D: coronary artery disease denome-wide replication and meta-analysis plus the coronary artery disease (c4d) genetics; OCAC: ovarian cancer association consortium; PRACTICAL: prostate cancer association group to investigate cancer-associated alterations in the genome; ILCCO: international lung cancer consortium; GAME-OA: Genetic Associations and Mechanisms in Oncology. FinnGen: FinnGen database(https://www.finngen.fi/en); COVID-19 hg7: version 7 data published by the COVID-19 Host Genetics Program(https://www.covid19hg.org/results/r7/); UKB: The UK Biobank(https://www.ukbiobank.ac.uk/).

Table S7. Mendelian randomization studies included in the meta-analyses of genetic liability to problematic alcohol use in relation to Circulatory system diseases, Digestive system diseases, Nervous system diseases and mental and behavioral disorders, Neoplasms, and Other diseases.

Diseases	Data sources	Cases	Controls	NIVs	MR-PRESSO		IVW			Weight Median		MR-Egger			Author, years
					P-gt	Outliers	P-Q	OR(95%CI)	P	OR(95%CI)	P	OR(95%CI)	P	P intercept	
**Diseases of the circulatory system**															
Heart failure	FinnGen	27304	349973	19	0.122	NA	0.125	0.74（0.56,0.99）	0.041	0.80（0.57,1.13）	0.206	0.83（0.22,3.11）	0.787	0.867	De novo MR; 2023
Peripheral vascular disease	FinnGen	2230	349539	20	0.260	NA	0.278	1.14（0.51,2.55）	0.741	0.92（0.32,2.65）	0.875	0.47（0.01,19.68）	0.697	0.639	De novo MR; 2023
Coronary artery atherosclerosis	FinnGen	47550	313400	17	<0.001	3	<0.001	0.89（0.61,1.29）	0.525	0.99（0.72,1.37）	0.965	0.98（0.17,5.67）	0.982	0.910	De novo MR; 2023
Stroke	FinnGen	25398	339920	18	0.025	1	0.011	1.24（0.88,1.76）	0.216	1.07（0.74,1.55）	0.703	0.59（0.09,3.86）	0.587	0.438	De novo MR; 2023
Myocardial infarction	FinnGen	24185	313400	18	0.001	1	0.001	0.86（0.56,1.32）	0.490	0.81（0.52,1.26）	0.342	2.12（0.31,14.57）	0.457	0.361	De novo MR; 2023

Atrial fibrillation	FinnGen	45766	191924	18	<0.001	2	<0.001	1.13 (0.68,1.88)	0.637	1.43 (0.98,2.07)	0.062	1.54 (0.14,16.87)	0.730	0.801	De novo MR; 2023
Hypertension	FinnGen	111581	265626	16	<0.001	4	<0.001	0.84 (0.50,1.40)	0.502	1.16 (0.89,1.52)	0.263	1.37 (0.12,14.87)	0.800	0.686	De novo MR; 2023
Venous thromboembolism	FinnGen	19372	357905	19	0.045	NA	0.053	1.30 (0.93,1.83)	0.123	1.13 (0.76,1.68)	0.549	0.43 (0.10,1.85)	0.272	0.145	De novo MR; 2023
Aortic aneurysm	FinnGen	7395	349539	19	0.150	NA	0.146	1.18 (0.71,1.94)	0.523	1.43 (0.77,2.68)	0.259	3.03 (0.31,29.49)	0.353	0.416	De novo MR; 2023
Varicose veins	FinnGen	29539	324121	19	0.001	1	<0.001	1.08 (0.75,1.58)	0.673	1.11 (0.79,1.58)	0.541	1.18 (0.20,6.87)	0.854	0.922	De novo MR; 2023
Diseases of the digestive system															
Cholangitis	FinnGen	1715	330903	20	0.246	NA	0.248	0.98 (0.39,2.46)	0.960	0.85 (0.24,2.93)	0.792	4.40 (0.06,308.18)	0.503	0.486	De novo MR; 2023
Cholelithiasis	FinnGen	37041	330903	15	<0.001	5	<0.001	1.26 (0.63,2.54)	0.515	1.24 (0.86,1.77)	0.252	8.29 (0.16,416.94)	0.309	0.356	De novo MR; 2023
Cholecystitis	FinnGen	4299	330903	19	0.006	NA	0.005	1.82 (0.82,4.02)	0.138	2.88 (1.22,6.77)	0.015	2.94 (0.08,114.43)	0.571	0.796	De novo MR; 2023

NAFLD	FinnGen	2275	375002	18	0.032	1	0.025	0.93 (0.34,2.51)	0.880	1.83 (0.61,5.54)	0.283	0.02 (0.00,1.70)	0.104	0.103	De novo MR; 2023
Alcoholic Liver Disease	FinnGen	2761	366450	19	0.254	NA	0.213	4.26 (1.98,9.19)	2.14E-04	3.80 (1.35,10.65)	0.011	3.47 (0.10,122.38)	0.503	0.909	De novo MR; 2023
Cirrhosis	FinnGen	3970	373307	19	0.011	NA	0.007	2.76 (1.22,6.22)	0.015	2.17 (0.91,5.20)	0.081	3.05 (0.07,133.95)	0.571	0.958	De novo MR; 2023
GERD	FinnGen	26184	320387	19	0.002	NA	<0.001	1.10 (077,1.58)	0.589	1.00 (0.67,1.47)	0.984	0.60 (0.12,3.02)	0.540	0.455	De novo MR; 2023
Gastric Ulcer	FinnGen	5935	320387	20	0.206	NA	0.190	1.47 (0.87,2.46)	0.149	1.58 (0.83,3.00)	0.165	4.60 (0.43,49.32)	0.224	0.346	De novo MR; 2023
Duodenal Ulcer	FinnGen	3520	320387	19	0.125	NA	0.103	1.58 (0.76,3.29)	0.221	1.98 (0.85,4.62)	0.115	0.68 (0.02,19.83)	0.827	0.623	De novo MR; 2023
Acute Gastritis	FinnGen	2370	320387	19	0.035	1	0.030	1.53 (0.58,4.04)	0.388	1.05 (0.34,3.29)	0.925	1.10 (0.01,98.98)	0.966	0.885	De novo MR; 2023
Chronic Gastritis	FinnGen	3320	330903	20	0.827	NA	0.534	1.41 (0.98,2.04)	0.063	1.28 (0.76,2.17)	0.351	0.75 (0.14,3.96)	0.736	0.452	De novo MR; 2023

Acute Pancreatitis	FinnGen	6223	330903	19	0.535	NA	0.507	2.41（1.52,3.82）	2.00E-04	1.80（0.91,3.56）	0.092	4.29（0.54,4.24）	0.187	0.583	De novo MR; 2023
Chronic Pancreatitis	FinnGen	3320	330903	20	0.827	NA	0.810	2.67（1.45,4.91）	0.002	2.51（1.11,5.67）	0.027	1.21（0.07,19.55）	0.895	0.575	De novo MR; 2023
Acute Appendicitis	FinnGen	28745	346283	19	0.020	NA	0.011	1.12（0.82,1.52）	0.470	1.22（0.86,1.73）	0.269	0.67（0.17,2.71）	0.583	0.471	De novo MR; 2023
Diverticular Disease	FinnGen	30649	301931	20	0.036	1	0.037	1.21（0.91,1.61）	0.195	1.07（0.78,1.45）	0.686	0.76（0.20,2.85）	0.687	0.488	De novo MR; 2023
Ulcerative Colitis	FinnGen	5034	371530	19	0.029	NA	0.037	1.17（0.61,2.27）	0.639	1.17（0.52,2.62）	0.707	5.08（0.26,99.04）	0.298	0.334	De novo MR; 2023
Noninfective enteritis and colitis	FinnGen	7988	359927	20	0.855	NA	0.875	1.09（0.73,1.62）	0.670	0.99（0.58,1.70）	0.971	1.53（0.25,9.23）	0.648	0.708	De novo MR; 2023
IBS	FinnGen	9323	301931	20	0.040	1	0.055	0.99（0.62,1.57）	0.963	1.03（0.60,1.76）	0.916	0.52（0.06,4.39）	0.557	0.554	De novo MR; 2023
Chronic Periodontitis	FinnGen	4434	259234	19	0.289	NA	0.258	1.76（0.97,3.19）	0.062	1.62（0.78,3.35）	0.194	0.87（0.06,13.23）	0.921	0.609	De novo MR; 2023
Mental and behavioral disorders															

Depression	FinnGen	43280	329192	18	<0.001	2	<0.001	1.30 (0.94,1.78)	0.108	1.28 (0.91,1.80)	0.161	1.02 (0.24,4.30)	0.980	0.739	De novo MR; 2023
ADHD	FinnGen	462	371117	15	<0.001	5	<0.001	0.90 (0.54,1.50)	0.693	1.18 (0.90,1.53)	0.225	0.96 (0.09,10.79)	0.976	0.958	De novo MR; 2023
Insomnia	FinnGen	4214	3514	20	0.360	NA	0.386	1.44 (0.82,2.52)	0.199	1.42 (0.67,3.02)	0.366	1.15 (0.08,15.72)	0.919	0.862	De novo MR; 2023
Diseases of the nervous system															
Epilepsy	FinnGen	11740	287837	19	0.010	1	0.022	0.99 (0.63,1.54)	0.950	0.92 (0.57,1.50)	0.752	0.89 (0.11,6.98)	0.911	0.920	De novo MR; 2023
Alzheimer's Disease	FinnGen	6489	170429	19	0.287	NA	0.263	1.24 (0.71,2.18)	0.621	1.20 (0.58,2.50)	0.621	0.12 (0.01,1.22)	0.091	0.059	De novo MR; 2023
Parkinson's Disease	FinnGen	4235	373042	19	0.140	NA	0.128	1.19 (0.62,2.30)	0.605	1.42 (0.65,3.12)	0.382	0.88 (0.04,18.79)	0.937	0.847	De novo MR; 2023
Multiple Sclerosis	FinnGen	2182	373987	20	0.881	NA	0.866	0.80 (0.38,1.70)	0.563	0.85 (0.30,2.41)	0.763	0.15 (0.01,4.53)	0.291	0.338	De novo MR; 2023
ALS	MRC-IEU	22040	62644	4	0.677	NA	0.379	0.98 (0.93,1.05)	0.595	0.98 (0.92,1.04)	0.497	0.98 (0.90,1.07)	0.719	0.937	De novo MR; 2023

Migraine	FinnGen	18477	15905	18	<0.001	3	<0.001	1.02 (0.64,1.60)	0.948	1.05 (0.65,1.69)	0.850	0.61 (0.07,5.00)	0.649	0.631	De novo MR; 2023
Normal-pressure hydrocephalus	FinnGen	767	375610	20	0.679	NA	0.701	0.64 (0.18,2.27)	0.488	0.37 (0.06,2.16)	0.271	0.02 (0.00,7.72)	0.220	0.266	De novo MR; 2023
Neoplasms															
Colorectal cancer	FinnGen	6509	287137	20	0.133	NA	0.140	0.98 (0.58,1.64)	0.938	0.82 (0.42,1.63)	0.579	6.93 (0.75,64.32)	0.106	0.095	De novo MR; 2023
Esophageal cancer	FinnGen	566	287137	19	0.314	NA	0.312	4.57 (0.91,22.83)	0.064	7.32 (0.85,63.26)	0.071	1163.88 (1.15,1173427.00)	0.062	0.126	De novo MR; 2023
Gastric cancer	FinnGen	1307	287137	20	0.747	NA	0.754	1.33 (0.50,3.50)	0.568	1.33 (0.33,5.47)	0.688	7.26 (0.09,594.88)	0.389	0.448	De novo MR; 2023
Hepatocellular carcinoma	FinnGen	453	287137	19	0.402	NA	0.447	1.85 (0.34,10.11)	0.477	4.96 (0.42,58.65)	0.204	9.50 (0.00,23643.48)	0.580	0.670	De novo MR; 2023
Pancreatic cancer	FinnGen	1416	287137	19	0.340	NA	0.312	0.42 (0.15,1.13)	0.086	0.58 (0.16,2.19)	0.423	0.09 (0.00,8.55)	0.315	0.509	De novo MR; 2023
Breast cancer	BCAC	133384	113789	26	0.721	NA	<0.001	1.03 (0.82,1.30)	0.781	0.91 (0.79,1.04)	0.173	0.85 (0.61,1.19)	0.347	0.141	Zhou et al; 2022

	FinnGen	15680	167189	18	<0.001	1	<0.001	0.86（0.46,1.59）	0.625	0.50（0.31,0.83）	0.007	0.15（0.01,2.54）	0.209	0.236	De novo MR; 2023
	Meta-anlysis	149064	280978					1.01（0.81,1.25）	0.947	0.71（0.40,1.26）	0.243	0.83（0.60,1.16）	0.271		
Ovarian cancer	FinnGen	1025	167189	19	0.126	NA	0.131	0.60（0.16,2.26）	0.450	0.75（0.14,3.97）	0.737	1.26（0.00,619.32）	0.942	0.812	De novo MR; 2023
Endometrial cancer	FinnGen	1967	1677	19	0.836	NA	0.828	0.61（0.27,1.40）	0.245	0.57（0.19,1.75）	0.329	1.77（0.04,75.38）	0.769	0.577	De novo MR; 2023
Cervical cancer	FinnGen	369	167189	19	0.791	NA	0.800	2.41（0.37,15.67）	0.357	6.13（0.49,76.42）	0.159	39.17（0.01,181963.40）	0.406	0.516	De novo MR; 2023
Prostate cancer	FinnGen	13216	119948	20	0.035	NA	0.033	1.07（0.68,1.67）	0.778	1.21（0.72,2.05）	0.472	1.03（0.13,8.23）	0.980	0.972	De novo MR; 2023
Bladder cancer	FinnGen	2053	287137	19	0.478	NA	0.466	0.50（0.22,1.12）	0.093	0.50（0.16,1.55）	0.233	3.05（0.08,113.06）	0.233	0.329	De novo MR; 2023
Kidney cancer	FinnGen	2223	287137	20	0.369	NA	0.341	1.30（0.59,2.85）	0.509	1.21（0.40,3.68）	0.739	6.62（0.19,236.51）	0.314	0.373	De novo MR; 2023

Brain cancer	FinnGen	764	287137	20	0.585	NA	0.561	1.84（0.52,6.51）	0.343	3.07（0.49,19.20）	0.231	2.27（0.01,693.90）	0.782	0.942	De novo MR; 2023
Head and neck cancer	FinnGen	2131	287137	20	0.618	NA	0.581	2.20（1.03,4.73）	0.042	2.43（0.83,7.11）	0.105	0.35（0.01,11.19）	0.559	0.299	De novo MR; 2023
Malignant melanoma	FinnGen	3960	286874	19	0.440	NA	0.411	1.24（0.69,2.23）	0.476	0.73（0.32,1.63）	0.441	0.52（0.04,7.76）	0.644	0.530	De novo MR; 2023
Non-Hodgkin lymphoma	FinnGen	928	287137	20	0.514	NA	0.494	1.41（0.45,4.46）	0.555	1.43（0.28,7.27）	0.668	48.66（0.26,9105..95）	0.163	0.191	De novo MR; 2023
Multiple myeloma	FinnGen	585	287129	19	0.309	NA	0.313	0.26（0.05,1.24）	0.092	0.34（0.04,2.88）	0.320	3370.55（4.14,2.74E+06）	0.030	0.011	De novo MR; 2023
Other diseases															
Pregnancy, childbirth and the puerperium															
Spontaneous abortion	FinnGen	16906	149622	19	0.397	NA	0.388	1.28（0.95,1.73）	0.104	1.13（0.73,1.74）	0.585	1.32（0.33,5.35）	0.698	0.965	De novo MR; 2023
Ectopic pregnancy	FinnGen	5648	149622	20	0.141	NA	0.113	0.92（0.53,1.62）	0.781	0.72（0.37,1.42）	0.345	2.29（0.17,30.95）	0.540	0.492	De novo MR; 2023
Puerperal sepsis	FinnGen	3940	202267	19	0.491	NA	0.534	0.99（0.55,1.76）	0.966	1.35（0.59,3.12）	0.477	0.23（0.02,3.09）	0.280	0.272	De novo MR; 2023

Diseases of the genitourinary system															
Hyperplasia of prostate	FinnGen	30066	119297	20	0.586	NA	0.569	0.99 (0.77,1.27)	0.941	1.14 (0.81,1.59)	0.463	0.67 (0.21,2.09)	0.493	0.493	De novo MR; 2023
Diseases of the musculoskeletal system and connective tissue															
Rheumatoid arthritis	FinnGen	9243	368029	19	0.574	NA	0.570	1.00 (0.68,1.46)	0.999	0.87 (0.50,1.52)	0.625	0.86 (0.15,4.85)	0.870	0.867	De novo MR; 2023
Low back pain	MRC-IEU	2439	460571	18	0.025	1	0.014	0.93 (0.68,1.28)	0.666	1.02 (0.72,1.44)	0.917	0.27 (0.07,0.99)	0.066	0.073	De novo MR; 2023
	FinnGen	29329	270964	19	0.013	1	0.019	0.92 (0.68,1.24)	0.592	1.01 (0.73,1.41)	0.932	0.29 (0.08,1.04)	0.075	0.087	De novo MR; 2023
	Meta-anlysis	31768	731535					0.93 (0.75,1.15)	0.493	1.02 (0.80,1.29)	0.893	0.28 (0.11,0.70)	0.006		
Osteoporosis	FinnGen	7300	358014	19	0.095	NA	0.092	1.09 (0.65,1.84)	0.742	1.11 (0.61,2.02)	0.733	1.05 (0.09,11.96)	0.971	0.973	De novo MR; 2023
Diseases of the skin and subcutaneous tissue															
Lupus erythematosus	FinnGen	652	353088	19	0.805	NA	0.770	0.98 (0.24,4.02)	0.980	0.88 (0.13,6.08)	0.897	0.13 (0.00,72.99)	0.531	0.525	De novo MR; 2023

Psoriasis	FinnGen	9267	364071	20	<0.001	1	0.001	1.18 (0.68,2.05)	0.560	1.34 (0.76,2.35)	0.309	0.47 (0.04,5.92)	0.564	0.473	De novo MR; 2023
Diseases of the respiratory system															
Asthma	FinnGen	42163	202399	16	<0.001	4	<0.001	1.23 (0.78,1.95)	0.374	1.60 (1.05,2.43)	0.027	0.45 (0.06,3.71)	0.472	0.355	De novo MR; 2023
Pneumonia	FinnGen	58174	319103	19	0.122	NA	0.109	1.23 (1.01,1.50)	0.042	1.17 (0.91,1.51)	0.214	0.57 (0.25, 1.30)	0.198	0.079	De novo MR; 2023
COVID-19 (severe)	HGI 7	18152	1145549	23	0.059	NA	0.049	1.18 (0.70,1.99)	0.525	0.91 (0.51,1.61)	0.751	0.46 (0.07,2.89)	0.418	0.306	De novo MR; 2023
URIT	FinnGen	69111	308166	18	0.001	1	0.014	1.14 (0.92,1.42)	0.238	1.06 (0.83,1.35)	0.635	0.98 (0.36,2.67)	0.970	0.767	De novo MR; 2023
Chronic rhinitis, nasopharyngitis and pharyngitis	FinnGen	10868	283342	20	0.004	1	0.002	0.90 (0.54,1.51)	0.700	0.58 (0.34,0.96)	0.036	0.13 (0.01,1.14)	0.082	0.089	De novo MR; 2023
Diseases of the eye and adnexa															
Senile cataract	FinnGen	59522	312864	19	0.012	NA	0.008	0.84 (0.64,1.11)	0.216	0.84 (0.61,1.16)	0.298	0.16 (0.06,0.44)	0.002	0.004	De novo MR; 2023
Glaucoma	FinnGen	18902	358375	18	0.011	1	0.023	0.77 (0.53,1.13)	0.195	0.80 (0.53,1.23)	0.309	2.45 (0.48,12.45)	0.296	0.172	De novo MR; 2023

Age-related macular degeneration	FinnGen	8913	348936	19	0.180	NA	0.193	1.02（0.65,1.62）	0.925	0.87（0.48,1.60）	0.662	0.39（0.05,3.16）	0.395	0.372	De novo MR; 2023
Diabetic retinopathy	FinnGen	10413	308633	18	<0.001	3	<0.001	0.89（0.38,2.10）	0.790	1.58（0.79,3.14）	0.191	1.74（0.03,98.60）	0.791	0.743	De novo MR; 2023

ADHD: Attention Deficit Hyperactivity Disorder syndrome; ALS: Amyotrophic lateral sclerosis; NAFLD: Nonalcoholic Fatty Liver Disease; GERD: Gastroesophageal reflux; IBS: irritable bowel syndrome ; URIT: Acute upper respiratory tract infection; ICD-10: International Classification of Diseases, Tenth Revision; NIVs: the number of Single Nucleotide Polymorphisms; P-gt: P-value tests for MR-PRESSO global pleiotropy; IVW: Multiplicative random-effects inverse-variance weighted; P-Q: p-value tests for the heterogeneity of Cochran's Q; OR: Odds ratio; CI: confidence interval; P-intercept: p-value tests for the intercept pleiotropy in MR-Egger; All statistical tests were two-sided. P < 0.05 was considered significant. MRC-IEU: medical research council integrative epidemiology unit; BCAC: the breast cancer association consortium. FinnGen: FinnGen database(https://www.finngen.fi/en); HGI 7: version 7 data published by the COVID-19 Host Genetics Program(https://www.covid19hg.org/results/r7/); UKB: The UK Biobank(https://www.ukbiobank.ac.uk/).

Table S8. MVMR analysis estimating the effect of alcohol consumption on disease, conditioning on smoking.

Diseases	Alcohol consumption		Smoking initiation	
	OR（95%CI）	P	OR（95%CI）	P
Epilepsy	1.12（0.87,1.43）	0.389	0.70（1.03,1.64）	0.03
Parkinson's Disease	0.76（0.64,0.91）	0.002	0.78（0.54,1.13）	0.188
Alcoholic Liver Disease	1.90（0.88,4.06）	0.099	1.91（1.16,3.15）	0.011
Cirrhosis	1.25（0.68,2.32）	0.471	2.04（1.34,3.10）	0.001
Duodenal Ulcer	1.47（0.92,2.35）	0.105	1.92（1.29,2.87）	0.001
Chronic Pancreatitis	1.98（1.14,3.44）	0.015	1.42（0.94,2.14）	0.096
Chronic Periodontitis	1.21（0.88,1.68）	0.247	1.48（1.03,2.12）	0.034
Spontaneous abortion	1.11（0.96,1.28）	0.152	1.33（1.10,1.61）	0.004
Hyperplasia of prostate	0.79（0.65,0.96）	0.018	1.16（0.94,1.43）	0.159
Rheumatoid arthritis	0.69（0.49,0.96）	0.028	1.28（0.97,1.69）	0.079
COVID-19（severe）	1.27（0.67,2.41）	0.457	1.27（1.33,3.08）	0.001
Stroke	1.11（0.81,1.51）	0.521	1.34（1.12,1.61）	0.001
Hypertension	1.05（1.00,1.10）	0.063	1.45（1.22,1.71）	＜0.001
Colorectal cancer	1.45（1.13,1.85）	0.004	0.82（0.60,1.13）	0.222

Esophageal cancer	1.75（0.80,3.82）	0.162	1.95（0.72,5.28）	0.185
Gastric cancer	1.89（0.90,3.94）	0.091	0.67（0.35,1.29）	0.228
Kidney cancer	0.83（0.49,1.40）	0.489	1.64（1.00,2.70）	0.052
Head and neck cancer	1.90（1.10,3.28）	0.021	1.37（0.81,2.30）	0.24

OR: Odds ratio; CI: confidence interval; All statistical tests were two-sided. $P < 0.05$ was considered significant.

Table S9. The effects model was corrected using the B/H correction.

Diseases	Alcohol consumption				Problematic alcohol use	
	Univariate MR		MVMR		Univariate MR	
	Original P-value	B/H corrected P-value	Original P-value	B/H corrected P-value	Original P-value	B/H corrected P-value
Epilepsy	0.014	0.079	0.389	0.493	0.95	0.993
Parkinson's Disease	0.005	0.044	0.002	0.038	0.605	0.897
Alcoholic Liver Disease	9.78E-07	3.86E-05	0.099	0.181	2.14E-04	0.008
Cirrhosis	4.00E-04	0.008	0.471	0.516	0.015	0.274
Duodenal Ulcer	0.001	0.013	0.105	0.181	0.221	0.701
Acute Pancreatitis	0.18	0.423	NA	NA	2.00E-04	0.008
Chronic Pancreatitis	0.003	0.03	0.015	0.08	0.002	0.049
Chronic Periodontitis	0.001	0.013	0.247	0.335	0.062	0.519
Spontaneous abortion	0.006	0.047	0.152	0.237	0.104	0.563
Hyperplasia of prostate	0.009	0.059	0.018	0.08	0.941	0.993
Rheumatoid arthritis	0.002	0.023	0.028	0.089	0.999	0.999
Pneumonia	0.796	0.898	NA	NA	0.042	0.438
COVID-19（severe）	0.049	0.188	0.457	0.516	0.525	0.897
Heart failure	0.398	0.699	NA	NA	0.041	0.438

Stroke	0.028	0.138	0.521	0.521	0.216	0.701
Hypertension	0.043	0.179	0.063	0.15	0.502	0.897
Colorectal cancer	0.013	0.079	0.004	0.038	0.938	0.993
Esophageal cancer	0.017	0.09	0.162	0.237	0.064	0.519
Gastric cancer	0.035	0.163	0.091	0.181	0.568	0.897
Lung cancer	2.00E-04	NA	NA	NA	NA	NA
Oral and oropharyngeal cancer	5.64E-13	NA	NA	NA	NA	NA
Kidney cancer	0.043	0.179	0.489	0.516	0.509	0.897
Head and neck cancer	0.007	0.05	0.021	0.08	0.042	0.438

MVMR: Genetic predictors of "alcohol consumption" and "smoking initiation" were adjusted for confounding; OR: Odds ratio; CI: confidence interval; All statistical tests were two-sided. P < 0.05 was considered significant.

Table S10. Search Strategies for PubMed, Embase, Web of Science, and Scopus Databases.

#1	Alcohol Consumption	Mesh：Alcohol Drinking
		Free terms：“Drinking,Alcohol”,“Alcohol Consumption”,“Consumption, Alcohol”, “Alcohol Intake”,“Intake, Alcohol”,“Alcohol Drinking Habits”,“Alcohol Drinking Habit”,“Drinking Habit, Alcohol”,“Habit, Alcohol Drinking”
#2	Genome-Wide Association Study	Mesh：“Genome-Wide Association Study”
		Free terms：“Association Studies, Genome-Wide”,“Association Study, Genome-Wide”,“Genome-Wide Association Studies”,“Studies, Genome-Wide Association”,“Study, Genome-Wide Association”,“Whole Genome Association Analysis”,“GWA Study”,“GWA Studies”,“Studies, GWA”,“Study, GWA”,“Whole Genome Association Study”,“Genome Wide Association Scan”,“Genome Wide Association Studies”,“Genome Wide Association Analysis”,“Genome Wide Association Study”
#3	Mendelian Randomization Analysis	Mesh: “Mendelian Randomization Analysis”
		Free terms：“Analysis, Mendelian Randomization”
#4	#2 OR #3	
#5	#1 AND #4	

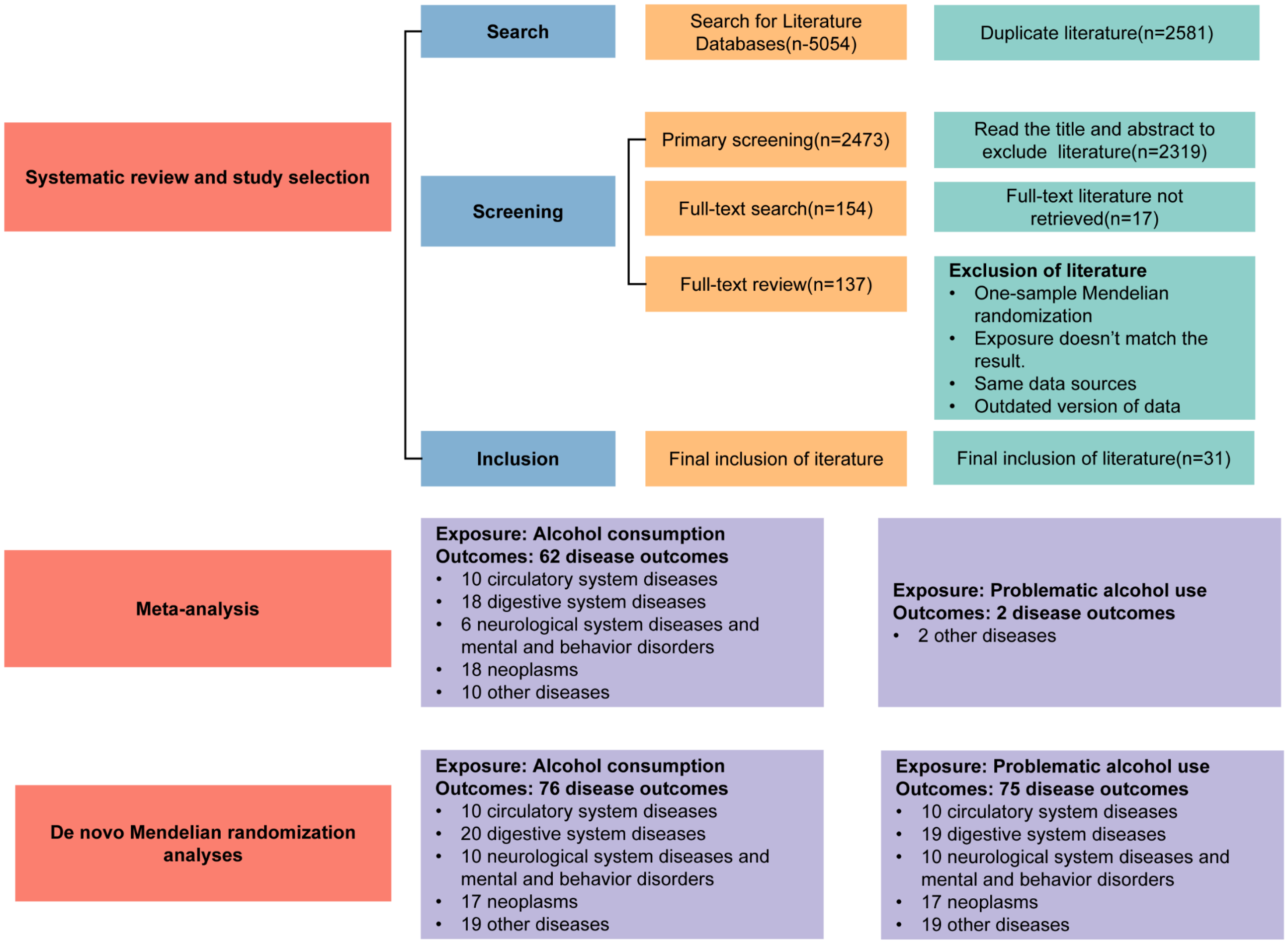

Figure S1. Literature review and study design.

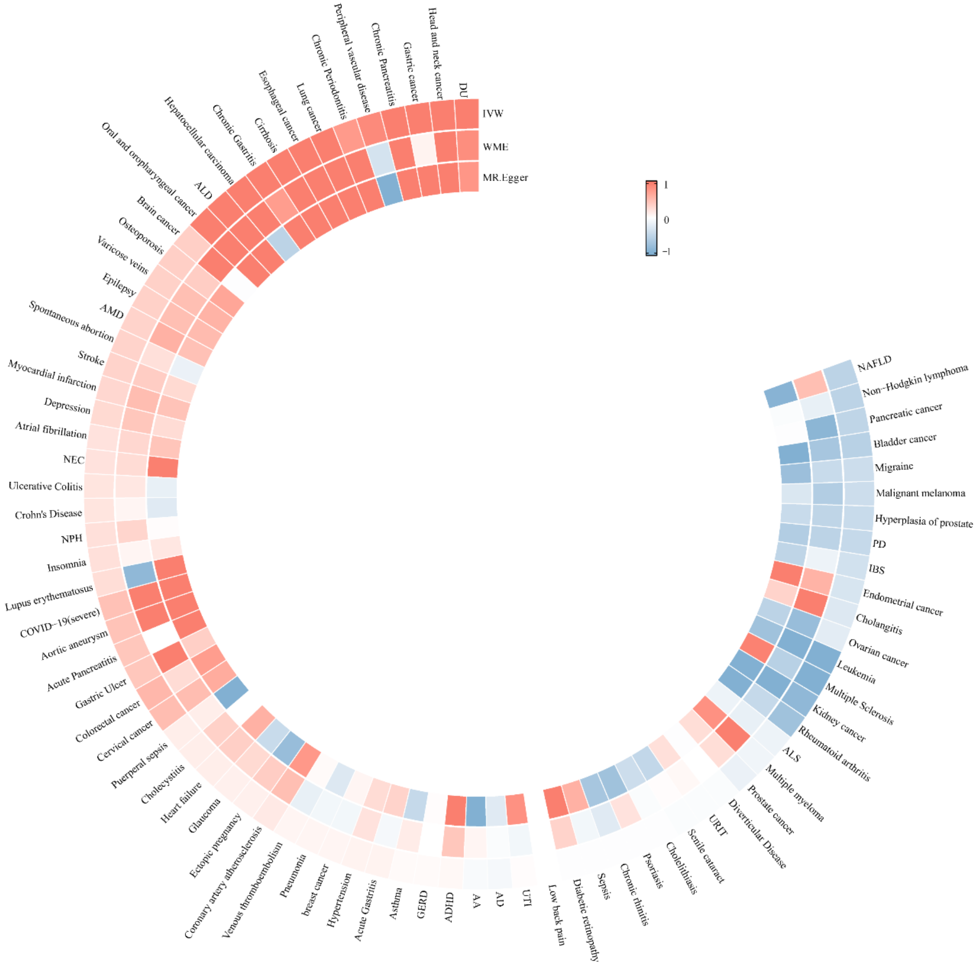

Figure S2. Genetic predictors of "alcohol consumption" and disease risk were summarized by IVW, WME, MR-Egger**, and analysis of beta values. beta values >0 were considered risk factors, and beta values <0 were considered protective factors.**

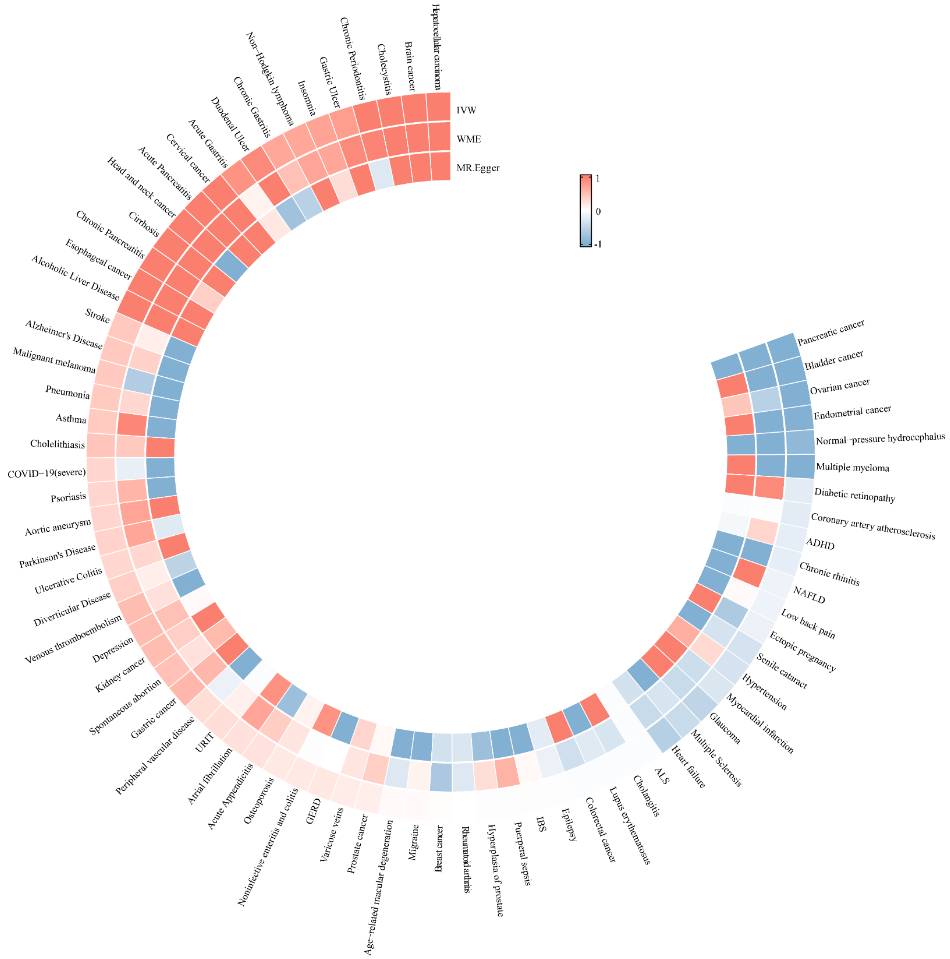

Figure S3. Genetic predictors of "problematic alcohol use" and disease risk were summarized by IVW, WME, MR-Egger, and analysis of beta values. beta values >0 were considered risk factors, and beta values <0 were considered protective factors.

Reference

1. Saunders, G.R.B.; Wang, X.; Chen, F.; Jang, S.K.; Liu, M.; Wang, C.; Gao, S.; Jiang, Y.; Khunsriraksakul, C.; Otto, J.M., et al. Genetic diversity fuels gene discovery for tobacco and alcohol use. *Nature* 2022, *612*, 720-724, doi:10.1038/s41586-022-05477-4.
2. Zhou, H.; Sealock, J.M.; Sanchez-Roige, S.; Clarke, T.K.; Levey, D.F.; Cheng, Z.; Li, B.; Polimanti, R.; Kember, R.L.; Smith, R.V., et al. Genome-wide meta-analysis of problematic alcohol use in 435,563 individuals yields insights into biology and relationships with other traits. *Nature neuroscience* 2020, *23*, 809-818, doi:10.1038/s41593-020-0643-5.
